# The Functional and Clinical Significance of the 24-Hour Rhythm of Circulating Glucocorticoids

**DOI:** 10.1210/er.2015-1080

**Published:** 2016-10-17

**Authors:** Henrik Oster, Etienne Challet, Volker Ott, Emanuela Arvat, E. Ronald de Kloet, Derk-Jan Dijk, Stafford Lightman, Alexandros Vgontzas, Eve Van Cauter

**Affiliations:** Medical Department I (H.O., V.O.), University of Lübeck, 23562 Lübeck, Germany; Institute for Cellular and Integrative Neuroscience (E.C.), Centre National de la Recherche Scientifique (CNRS) UPR 3212, University of Strasbourg, 67084 Strasbourg, France; Division of Endocrinology, Diabetology and Metabolism (E.A.), Department of Internal Medicine, University of Turin, 10043 Turin, Italy; Department of Endocrinology and Metabolic Disease (E.R.d.K.), Leiden University Medical Center, 2333 ZA Leiden, The Netherlands; Surrey Sleep Research Center (D.-J.D.), Faculty of Health and Medical Sciences, University of Surrey, Guildford GU2 7XP, United Kingdom; Henry Wellcome Laboratories for Integrative Neuroscience and Endocrinology (S.L.), University of Bristol, Bristol BS8 1TH, United Kingdom; Sleep Research and Treatment Center (A.V.), Department of Psychiatry, Pennsylvania State University College of Medicine, Hershey, Pennsylvania 17033; and Sleep, Metabolism, and Health Center (E.V.C.), Department of Medicine, University of Chicago, Chicago, Illinois 60637

## Abstract

Adrenal glucocorticoids are major modulators of multiple functions, including energy metabolism, stress responses, immunity, and cognition. The endogenous secretion of glucocorticoids is normally characterized by a prominent and robust circadian (around 24 hours) oscillation, with a daily peak around the time of the habitual sleep-wake transition and minimal levels in the evening and early part of the night. It has long been recognized that this 24-hour rhythm partly reflects the activity of a master circadian pacemaker located in the suprachiasmatic nucleus of the hypothalamus. In the past decade, secondary circadian clocks based on the same molecular machinery as the central master pacemaker were found in other brain areas as well as in most peripheral tissues, including the adrenal glands. Evidence is rapidly accumulating to indicate that misalignment between central and peripheral clocks has a host of adverse effects. The robust rhythm in circulating glucocorticoid levels has been recognized as a major internal synchronizer of the circadian system. The present review examines the scientific foundation of these novel advances and their implications for health and disease prevention and treatment.

## ESSENTIAL POINTS

- The daily rhythmicity of plasma glucocorticoid (GC) levels is a strong modulator of many physiological and psychological processes, although its functional significance is poorly understood.
- The suprachiasmic nuclei of the hypothalamus have been shown to harbor a molecular clock mechanism generating circadian rhythmicity in mammals, but the same mechanism is present in many peripheral tissues and elsewhere in the brain.
- Mineralocorticoid receptors and glucocorticoid receptors mediate the action of naturally occurring GC in complementary fashion.
- Optimal physiological effects of GC occur when the central signal that controls the rhythm of GC release and the peripheral rhythms in tissues expressing GC receptors are aligned.
- New studies suggest that misalignment of central and peripheral oscillators may increase the risk of disease, with adverse effects on the immune system, cardiovascular system and metabolism, among others prominent.
- Chronopharmacological strategies that attempt to normalize the rhythm of circulating GCs have potential to improve the treatment of a wide variety of physical and mental conditions.

## I. Introduction

### A. Previous understanding of circadian rhythms

Since the discovery of the 24-hour variations of excreted ketosteroids by G. Pincus in the early 1940s (1) and later of the daily rhythm of plasma glucocorticoid (GC) levels in both humans and laboratory rodents (2, 3), an abundant literature has documented that the daily variation of circulating GCs is perhaps the largest and most robust circadian rhythm of all blood constituents in mammals. Although the multiple actions of GCs on immunity, inflammation, metabolism, cognitive function, mood, growth, reproduction, and cardiovascular function and on the stress response (see *Box 1*) are well recognized, the functional significance of the wide daily variation of their circulating levels for these multiple functions and for the circadian timing system itself has only begun to be understood. As will be detailed below, daily rhythmicity is regulated at different levels of the hypothalamo-pituitary-adrenal (HPA) axis. In turn, circulating GCs are internal time-givers that can affect the circadian organization of many peripheral organs as well as of multiple brain regions. In humans, the main GC is cortisol, whereas corticosterone circulates in a 10-fold lower concentration. In contrast, in rodents, corticosterone is the primary GC.

Box 1. **The Stress Concept**

**Stress was defined by Hans Selye** **(4)** **as “a state of nonspecific tension in living matter, which manifests itself by tangible morphologic changes in various organs and particularly in the endocrine glands which are under anterior pituitary control.” This “state of stress” is evoked by a stressor, which is defined as any stimulus that disrupts cellular “homeostasis” or, on the organismic level, as “a real or interpreted threat to the physiological and psychological integrity”** **(5, 6)****.**

**Processing of stressful information occurs in the central nervous system via different pathways. Physical (sensory) stressors (heat, cold, injury, immune activation) are communicated directly from the brain stem to the hypothalamus. Psychological stressors either real or imagined are processed in higher brain regions and reach the hypothalamus via multisynaptic pathways. In the hypothalamic paraventricular nucleus (PVN), the processed stressful information activates the sympathetic nervous system and HPA axis, which coordinate the organism’s defense reactions with the goal to restore homeostasis and to promote (behavioral) adaptation. The principal conductor of the stress response is corticotropin-releasing hormone (CRH) together with a cocktail of secretagogues, notably vasopressin. The activation of the HPA axis and its GC end-product occurs over a background of circadian and ultradian variations** **(7)****.**

**GCs are secreted late in the stress response and usually reach maximum levels after 15 minutes of exposure. “Glucocorticoids acting via glucocorticoid receptors (GR) prevent initial stress reactions (eg, autonomic, immune, inflammatory, metabolic, brain) from overshooting and becoming damaging themselves”** **(8)****. The action via mineralocorticoid receptors (MRs) is opposite and enhances the initial stress reaction** **(9, 10)** **(see** **Box 2****).**

***Coping.* Some researchers rather restrict the definition of stress entirely to a psychological construct because every stressor, even the physical ones, is also processed in higher brain regions “under conditions where an environmental demand exceeds the regulatory and adaptive capacity of an organism, in particular in case of unpredictability and uncontrollability”** **(11)****. The most stressful condition is: no information, no control, and no prediction of upcoming events, with an uncertain feeling of threat. A safe place, social context, and self-esteem help one to cope** **(12)****. It is not so much what happens but rather how the individual interprets the situation and copes. It emphasizes the importance of anticipation and appraisal of important information.**

***Allostasis.* The concept of “allostasis” was introduced to describe the condition when structure and function of brain networks adapt to or even anticipate real or perceived changes in the environment** **(6)****. Allostasis generally occurs as a result of cumulative stress exposure and describes an unstable equilibrium characterized by variable set points, as opposed to homeostasis where return to stability is the ultimate goal. The term “allostatic load” designates the cost of allostasis through energyconsuming adaptations** **(13)****.**

**Figure 1.**
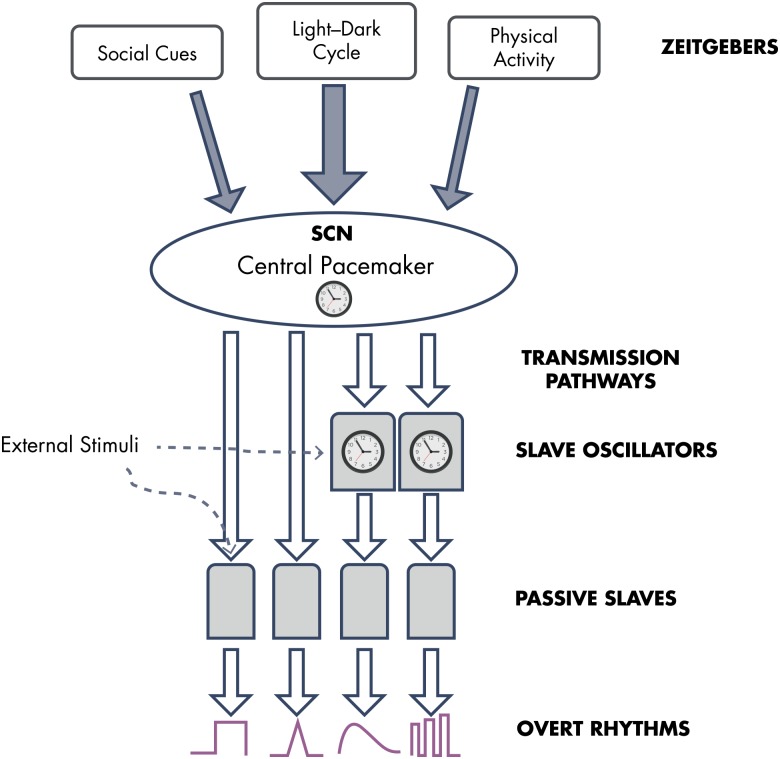
Schematic conceptual representation of the mammalian circadian system as it was understood circa 1990. A circadian pacemaker located in the SCN of the hypothalamus was widely thought to be the only self-sustained 24-hour clock and to act as master pacemaker for the entire organism. The molecular mechanism generating the self-sustained central circadian signal was not known. The light-dark cycle had been recognized as the main external synchronizer (zeitgeber) of the master pacemaker, but inputs from social cues and physical activity had also been identified. A few peripheral tissues—including the adrenals—had been found to generate damped oscillations that require input from the master pacemaker to be sustained (“slave” oscillators). Most peripheral tissues were considered not capable of generating self-sustained circadian oscillations and were thought to be passively entrained by the master pacemaker (“passive slaves”). The 24-hour rhythm of circulating GCs was seen as an “overt” rhythm reflecting central circadian timing, with inputs modifying its wave shape elicited by external and internal stimuli. [Redrawn and modified from E. Van Cauter and J. Aschoff: Endocrine and other biological rhythms. In: DeGroot LJ, ed. *Endocrinology.* Vol 3. WB Saunders; Philadelphia, PA: 1989; 2658–2705 (19), with permission.]

Circadian rhythms (from Latin “circa” meaning “about,” and “dies” meaning “day”) in physiological and behavioral processes are a ubiquitous feature in living organisms. Circadian rhythms are not just a passive response to daily cyclic fluctuations in the environment, but instead rely on endogenous, self-sustained clocks and, thus, persist under constant environmental conditions. In the literature, several terms are often used interchangeably when referring to variations with a period of close to 24 hours. For the sake of clarity, in the present review, we will restrict the use of the term “circadian” to rhythms controlled, at least partly, by endogenous clocks. When the origin of a rhythm is clearly exogenous or its control by an endogenous clock mechanism is still unproven, we will use the term “daily rhythm” or “24-hour rhythm.” Lastly, we will use the term “diurnal” to indicate “daytime” as opposed to “nighttime” or “nocturnal” as, for example, to refer to “diurnal” vs “nocturnal” species. Clock-controlled mechanisms allow anticipatory temporal organization of biological functions according to predictable changes (eg, the daily light-dark cycle) and, just as importantly, a temporal segregation between conflicting or incompatible processes (eg, feeding and sleeping). After pioneer studies showing that bilateral damage to the suprachiasmatic nuclei (SCN) of the hypothalamus results in the disappearance of daily rhythmicity of plasma corticosterone, drinking behavior, and rest-activity in rats (14, 15), much experimental work has confirmed that the SCN harbors a self-sustained clock playing an essential role for the maintenance of circadian rhythmicity in mammals. Even when isolated from the rest of the brain, the SCN clock continues to oscillate (16). Furthermore, transplantation of fetal SCN cells can restore the rest-activity rhythm in adult animals rendered arrhythmic by SCN lesions (17). For a while, the SCN was thus viewed as the single self-sustained pacemaker that drives internal rhythmicity, entraining “slave” oscillators in other tissues (18). A schematic representation of the view of the mammalian circadian system that prevailed until the early 1990s is given in Figure 1 (19).

### B. Recent advances and implications for health and disease

**Figure 2.**
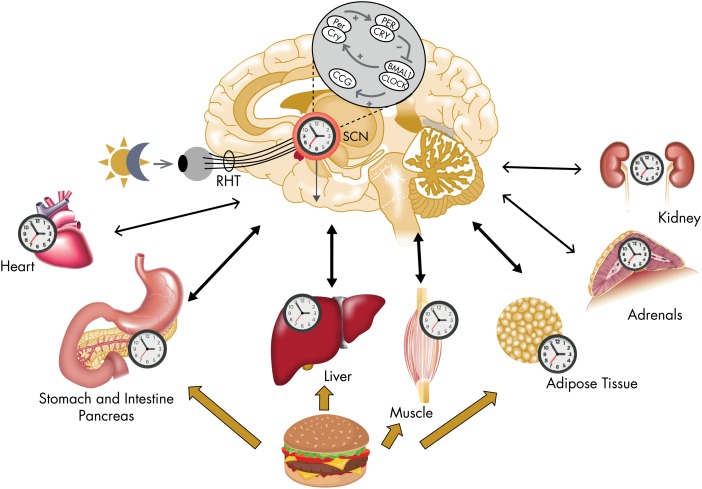
Illustration of the mammalian circadian system as conceptualized circa 2010. The molecular mechanism generating a self-sustained circadian oscillation in SCN neurons is a complex transcriptional-translational feedback loop comprising core transcriptional activators BMAL1/CLOCK and two sets of repressors PER and CRY. The core transcriptional activators BMAL1/CLOCK regulate numerous genes, referred to as “clock-controlled genes” (CCGs).The same molecular machinery has been found in other central tissues as well as in nearly all peripheral tissues examined so far. The light-dark cycle is the main external synchronizer of the central circadian pacemaker (via the retino-hypothalamic tract [RHT]), but other external stimuli can affect the phase and amplitude of peripheral oscillators. In particular, the timing of food intake has been recognized as an important external synchronizer for circadian time-keeping in peripheral organs involved in the control of energy metabolism. The master clock in the SCN serves to synchronize central and peripheral oscillators to optimize the function of the organism relative to the 24-hour periodicities in the environment. Signals from peripheral tissues can affect the phase and amplitude of the central pacemaker. [Elements of this figure were published in M. Garaulet and J. A. Madrid: Chronobiological aspects of nutrition, metabolic syndrome and obesity. *Adv Drug Deliv Rev.* 2010;62(9–10):967–978 (20), with permission. © Elsevier.]

Starting around the turn of the millennium, an increasing amount of experimental evidence has challenged the monopoly of the SCN clock as endogenous circadian pacemaker. In particular, it has been shown that many peripheral tissues are capable of generating self-sustained oscillations when isolated in vitro and can be synchronized by external or internal cues, independently of the master SCN clock. Therefore, the physiological regulation of circadian rhythms is now thought to involve a multioscillatory system, comprising a master circadian pacemaker in the SCN and numerous secondary clocks in other cerebral regions and in most peripheral organs, including endocrine glands (20). Figure 2 illustrates the contemporary view of the mammalian circadian system. The molecular mechanisms generating circadian rhythmicity within pacemaker neurons of the SCN have been identified as a complex transcriptional-translational feedback loop. The same interacting molecular circuitry of core clock elements and clock-dependent elements is present in multiple, if not all, peripheral tissues, including heart, kidney, muscle, liver, pancreas, blood, and fat. Specifically, confirming old suspicions (21), a circadian clock has been newly discovered in the adrenal glands and has been shown to participate in the control of the 24-hour rhythm of GCs (22), thereby revealing an additional element of circadian control to the regulation of the HPA axis.

**Figure 3.**
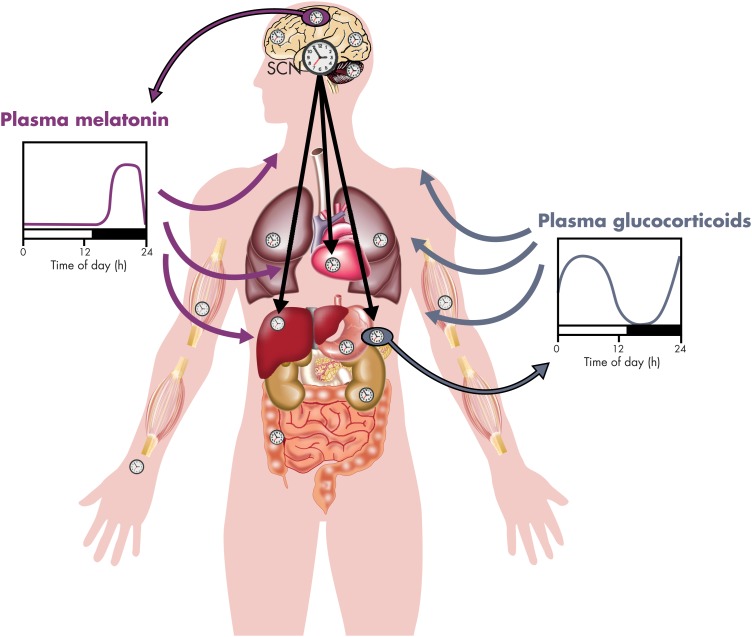
Schematic representation of the pathways involved in the internal synchronization of the central suprachiasmatic (SCN) clock with the peripheral oscillators in the human circadian system. Both direct neural signals (transmitted by the autonomous nervous system, represented by black arrows) and indirect hormonal signals are involved. The 24-hour rhythms of circulating melatonin (released by the pineal gland, represented by a purple circle) and cortisol (released by the adrenals, represented by a blue circle) are considered as primarily controlled by the central SCN clock. In the schematic representations of the melatonin and GC profiles, the black bars represent the sleep/dark period. The blue and purple arrows symbolize, respectively, the synchronizing effects of the GC and melatonin rhythms. Because of the ubiquity of GRs in the entire organism, the 24-hour rhythm of circulating GCs plays a major role in synchronizing central and peripheral clocks. [Modified and redrawn from P. Pevet and E. Challet: Melatonin: both master clock output and internal time-giver in the circadian clocks network. *J Physiol Paris.* 2011;105(4–6):170–182 (99), with permission. © Elsevier.]

Although the environmental light-dark cycle is the primary external synchronizer, or zeitgeber, of the master clock in the SCN, the timing of food intake and fasting has lately been recognized as a potent synchronizer of peripheral clocks (Figure 2). The central SCN clock regulates behavioral rhythms, including the sleep-wake and feeding cycles, and also entrains peripheral clocks via neural and humoral mechanisms, including the activity of the two branches of the autonomous nervous system and the rhythmic release of GCs and melatonin (illustrated in Figure 3). A regular 24-hour temporal organization is thought to be important for good health. Laboratory studies manipulating circadian organization in both humans (23–25) and rodents (26, 27) and a vast literature on the epidemiology of shift work (28–30) have documented that misalignment of central and peripheral clocks has adverse health consequences. Because GC receptors (nuclear receptor subfamily 3, group C, member 1, NR3C1, referred to as GR in the remainder of this text; see *Box 2*) are widely distributed both in the central nervous system and in the periphery, the robust rhythm of GC release can be viewed as a major internal synchronizing signal of the mammalian circadian system (31). In contrast, mineralocorticoid receptors (nuclear receptor subfamily 3, group C, member 2, NR3C2; referred to as MRs in the remainder of this text) no longer respond when levels of GC are elevated above basal values and therefore do not mediate circadian modulation of target tissue activity (see *Box 2*)*. A* corollary of this concept is that alterations in the 24-hour rhythm of GC levels may promote circadian misalignment and its adverse health consequences. Multiple studies have shown that the 24-hour profile of cortisol concentrations does not adapt rapidly to acute shifts in light-dark, activity-rest and/or feeding cycles, as occur in jet lag and shift work rotations, although its amplitude may be reduced under some conditions (32). Under such conditions, the misalignment of the 24-hour cortisol rhythm causes or exacerbates a lack of synchronization between central and peripheral clocks. Pathological conditions where cortisol production is either excessive or insufficient are associated with profound alterations of the 24-hour profile of peripheral concentrations. Under such conditions, the synchronizing signal provided by the 24-hour rhythm of GC concentrations is disrupted, potentially leading to a reduction in the overall alignment of the circadian system and adverse health consequences. For example, in patients with adrenal insufficiency, the failure to restore a normal GC profile by current regimens of replacement therapy may be partly responsible for the doubling of the age-adjusted mortality risk (33) and for the severe mental and physical fatigue with many patients remaining unfit for work (34). Chronopharmacological strategies that attempt to normalize the rhythm of circulating GCs may thus help to improve the efficacy of treatment and prevent or reduce complications.

Box 2. **Mineralocorticoid Receptors and Glucocorticoid Receptors**

***Properties.* The MR (nuclear receptor subfamily 3, group C, member 2, NR3C2) has a 10-fold higher affinity for the naturally occurring GCs (cortisol and corticosterone) than the GR (nuclear receptor subfamily 3, group C, member 1, NR3C1). The MR is not a selective receptor because it can also bind with high affinity aldosterone and progesterone** **(35, 36)****. Aldosterone selectivity in epithelial cells (kidney, intestine, salivary gland) is conferred by the oxidase 11*β*-hydroxycorticosteroid-dehydrogenase type 2 (HSD-2)** **(37)****. In the brain, these aldosterone-selective MRs are restricted to the nucleus tractus solitarius and the circumventricular organs** **(38)****. MR is also expressed abundantly in hippocampal pyramidal and dentate gyrus neurons, lateral septum, amygdala, and cingulate neurons (limbic structures), but here the naturally occurring GCs are the principal ligands because of their 100- to 1000-fold excess over aldosterone** **(39)****. Moreover, neurons express the reductase HSD-1, which regenerates bioactive GCs. Because of the 10-fold lower affinity of GRs, these receptors only become occupied when GC levels are elevated after stress or are near their circadian peak** **(35)****. In the brain, the GR is widely expressed in neurons and glial cells, and most abundantly in typical stress-regulating centers (eg, PVN of the hypothalamus, limbic structures, frontocortical regions, and ascending aminergic neurons)** **(40)****. The GR is nearly ubiquitous in peripheral tissues, whereas the distribution of the MR is much more limited. Synthetic GCs (dexamethasone, prednisolone) are ligands for GR. *Molecular mechanisms.* MR and GR are ligand-driven transcription factors involved in the regulation of gene expression in interaction with other transcription factors and coregulators. Recently, both receptor types were also found to rapidly regulate excitability of neurons. MR increases excitability by enhancing the release of excitatory transmitters** (41–43)**. GR rapidly promotes the release of endocannabinoids that trans-synaptically attenuate transiently raised excitability** **(44)****.**

***Functions.* MR and GR mediate in complementary fashion the action of the naturally occurring GC. MR operates during the initial phase of the stress response by facilitating appraisal processes and the selection of an appropriate coping response** (45–47)**. Subsequently, with rising GC levels, GR promotes negative feedback in the HPA axis and behavioral adaptation, while facilitating the storage of the experience in memory networks for future use** **(9, 48)****. When visualized by functional magnetic resonance imaging, the initial phase of the stress response is characterized by increased activation of the limbic network (to appraise the most vital information) at the expense of the frontocortical regions, which later become activated as part of executive control. MR manipulation demonstrates switching of circuits in the initial stress reaction, showing the crucial role of this receptor in the choice of behavioral coping style** **(49)****. On many (but not all) GC targets (inflammatory, immune, cognition, emotion), MR and GR mediate opposing actions, suggesting that the MR:GR balance is crucial for homeostasis/allostasis and adaptation** **(36)****.**

### C. Aims and structure of the review

The overall goals of the present review are to provide an update on the circadian organization of the mammalian system, to discuss the role of the 24-hour rhythm of circulating GCs as a major internal synchronizer of central and peripheral clocks, and to derive the implications of abnormalities of this rhythm for health and disease. The article will start with a brief description of how the multioscillatory circadian system drives rhythmicity of physiological processes in the brain and peripheral tissues and then summarize the current understanding of the control of the 24-hour rhythm of circulating GC levels and modulatory influences by external and internal factors. After a short overview of the multiple physiological actions of GCs, human conditions where abnormalities of the 24-hour profile of cortisol have been identified will be discussed, and their implications for cardiometabolic risk, central nervous system function, and quality of life will be extrapolated. Finally, we will examine the potential therapeutic implications of correcting or restoring this internal synchronizing signal.

## II. The Organization of the Mammalian Circadian System

### A. Central and peripheral circadian clocks

Rhythmic regulation of physiology and behavior on a daily basis is shared across phylogenetic kingdoms and relies on a complex system of cellular clocks with endogenous periods close to 24 hours. These cellular clocks are synchronized by multiple inputs, and their outputs deliver local as well as widely distributed temporal information. In mammals, circadian clocks are found in various brain and peripheral tissues, including the adrenal glands. Following the remarkable observation in 1998 that a serum shock can trigger persistent rhythmicity in cultured fibroblasts (50), a number of peripheral organs studied ex vivo have been found capable of maintaining self-sustained oscillations with a period approximating 24 hours, the main feature defining a circadian clock (51–53). Thus, as illustrated in Figure 2, the circadian clock in the SCN is not driving all peripheral rhythms, but rather acts as a master pacemaker for a multioscillatory circadian system (54, 55).

### B. Molecular mechanisms

**Figure 4.**
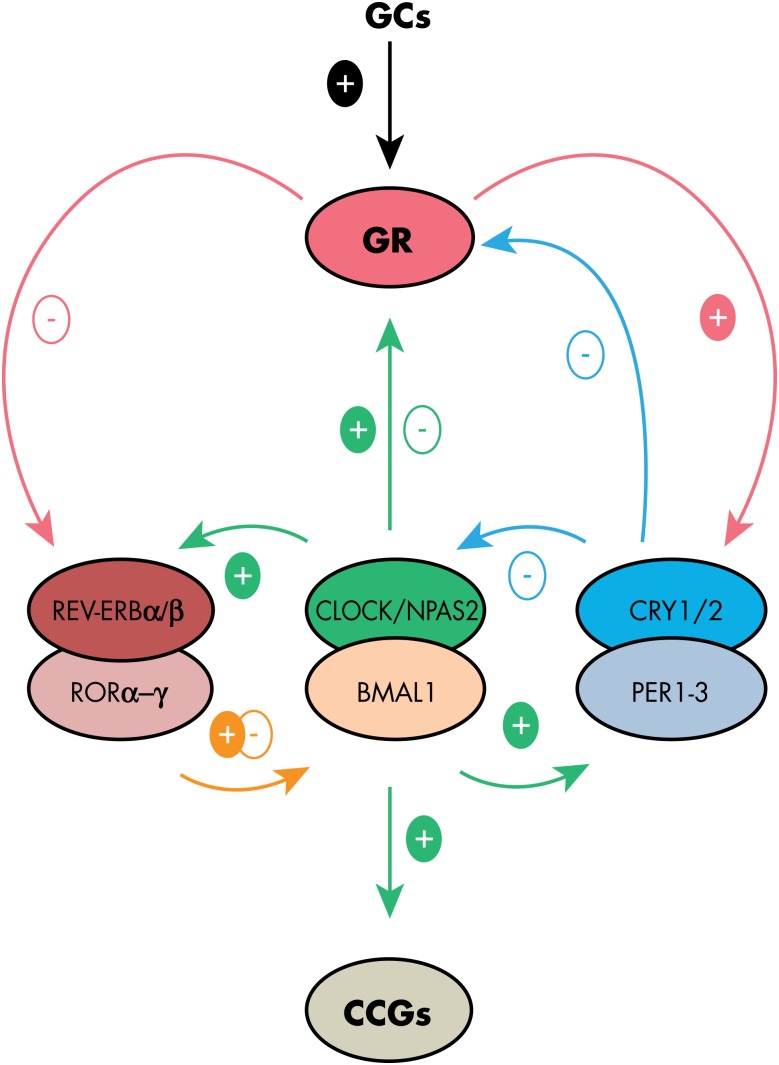
Molecular feedback loops generating circadian rhythmicity in both central and peripheral tissues. The upper part of the figure illustrates the interactions with GR activity. The basic transcription factors CLOCK (or its analog NPAS2) and BMAL1 heterodimerize and initiate the main positive loop by activation of other clock genes, including three *Period* genes (*Per 1*–*3*) and two *Cryptochrome* (*Cry 1*–*2*) genes. PER and CRY proteins form complexes that translocate to the nucleus where they inhibit their own CLOCK/BMAL1-induced transactivation, defining a main negative loop. CLOCK(NPAS2)/BMAL1 dimers also drive transcription of nuclear receptors of the REV-ERB and ROR families, including *Rev-erb α-β* (*Nr1d1*–*2*) and *Ror α-β-γ.* In turn, REVERBs and RORs inhibit and activate, respectively, the rhythmic transcription of *Bmal1* and *Clock.* Although GR activation can reset the phase of the clock by regulating Per expression and REVERB activity, the clock machinery modulates GR activity at transcriptional and post-translational levels in multiple tissues, thus gating the regulation of GC target genes in a tissue-specific fashion.

At the molecular level, central and peripheral circadian clocks are based on autoregulatory feedback loops involving so-called “canonical clock genes” and their protein products that mutually interact over a 24-hour cycle (Figure 4). The basic helix-loop-helix transcription factors circadian locomotor output cycles kaput (CLOCK; or its analog, neuronal PAS domain protein 2 [NPAS2]) and brain and muscle aryl hydrocarbon receptor nuclear translocator-like 1 (BMAL1) heterodimerize and initiate the main positive loop by activation of other clock genes, including three *Period* genes (*Per 1–3*) and two *Cryptochrome* genes (*Cry 1–2*)*.* PER and CRY proteins form complexes that translocate to the nucleus where they inhibit their own CLOCK/BMALl-induced transactivation, defining a main negative loop. The PER/CRY repressor complexes are inactivated by the proteasomal pathway (54, 55). This step is currently thought to be critical for allowing a new cycle of autoregulation to restart. CLOCK(NPAS2)/BMAL1 dimers also drive transcription of nuclear receptors of the reverse viral erythroblastosis oncogene product (REV-ERB) and retinoid-related orphan receptor (ROR) families, including *Rev-erb α-β* (*Nr1d1*–*2*) and *Ror α-β-y.* In turn, REV-ERBs and RORs inhibit and activate, respectively, the rhythmic transcription of *Bmal1* and *Clock* (54, 56). Other genes, such as *differentially expressed in chondrocytes 1*–*2* (*Dec* *1–2*)*,* as well as members of the PAR bZIP transcription factor family define further reinforcing loops within the molecular clockwork (57–60).

Besides its role as a transcriptional activator, CLOCK also has an acetyl-transferase activity driving the cyclic acetylation of various targets, including histones and BMAL1. Many other chromatin-modifying enzymes interact with the clock gene machinery. Among them, the histone deacetylase Sirtuin 1 (SIRT1) directly interacts with clock proteins (61). Post-translational changes, like methylation, phosphorylation, or ubiquitination, control the stability and cellular localization of the clock proteins, thus modulating the period of the circadian cycle (62).

Many other genes are also transactivated by CLOCK-(NPAS2)/BMAL1. These clock-controlled genes (Figure 4) are tissue-specific and generate intra- and intercellular signals that participate in the translation of temporal information originating from the clock into physiological functions (54). Among these is the gene *arginine vasopressin *(*Avp*), for which rhythmic transcription and translation in SCN neurons are controlled by the circadian clock (63). The circadian release of AVP from SCN cells provides temporal cues to the sub-paraventricular region, a main target of the master clock (64–66).

### C. External synchronizers

Among the environmental factors that impact on circadian timing, the light-dark cycle and the timing of food intake are the most powerful synchronizers (often referred to as zeitgebers, from the German “time giver”), although, as illustrated in Figure 2, they act at different levels of the circadian system. The major zeitgeber for the SCN is ambient light. More precisely, synchronization with the environmental light-dark cycle occurs by means of adjustment of the phase and period of the master clock every day. The term “phase” refers to the timing of a reference point (eg, the maximum or the minimum) on a periodic waveform. A phase shift is an advance or a delay of the timing of this reference point. Light-induced phase shifts depend on the time of the circadian cycle when light is perceived. The phase-response curve to brief light pulses presented over a background of constant darkness is characterized by phase advances during the late subjective night (ie, the end of the active period in nocturnal rodents and the end of the sleep period in humans) and phase delays during the early subjective night (67, 68). In humans, the phase delay region is generally longer than the phase advance region, and phase delays are on average larger than phase advances. In sum, the “average” human clock can be more easily phase delayed than phase advanced. Light is perceived by the retinae, which activate the SCN clock via direct retino-hypothalamic projections. Importantly, melanopsin, a newly identified photopigment sensitive to irradiance (ie, light intensity), is expressed in a subset of ganglion cells that specifically project to the SCN (69). These intrinsically photosensitive ganglion cells are essential for photo-entrainment (70, 71). Yet, the classical cone and rod photoreceptors of the outer retina, which are vital for the image-forming visual system, also play an indirect role in transmitting light conditions to the SCN (72, 73).

Meal timing, when imposed to experimental animals by temporally restricted feeding to a fairly narrow time window, is a potent synchronizer for most clocks in peripheral organs, including the liver, adipose tissue, muscle, gut, and pancreas. The demonstration that restricted feeding can uncouple the phase of peripheral clocks from the SCN, which stays phase-locked to the light-dark cycle (74, 75), has been a major breakthrough in this field because it showed that components of the circadian system can be synchronized independently of the master SCN pacemaker. Although the underlying mechanisms are not totally understood, meal-induced insulin and incretin hormone secretion could be an important mediator of entrainment of peripheral clocks by feeding (76–78). Feeding cues are efficient to adjust the timing of many circadian clocks and oscillators, not only at the periphery, but also within the brain of nocturnal rodents (79–82).

Only a few studies have investigated the effects of meal timing in diurnal (day-active) animals and in humans. Delaying food access to midnight in diurnal grass rats shifts their insulin pattern, but not their corticosterone profile, the latter being only phase-shifted when midnight feeding is hypocaloric (83). Morning meals rich in carbohydrates produce phase advances of circadian rhythms of body temperature and heart rate in subjects maintained awake and supine in bed for 48 hours under constant dim light (84). In contrast, the centrally controlled melatonin rhythm is not modified. These findings suggest that meal timing affects human peripheral clocks (84). Daytime fasting during the month of Ramadan has also been used to evaluate the effects of temporal restricted feeding in humans (for review, see Ref. 85). Daily rhythms of plasma leptin and possibly melatonin display phase delays (86, 87). The impact of Ramadan on plasma cortisol will be mentioned later (see *Section III*).

The light-entrainable clock in the SCN is only sensitive to nutritional cues associated with metabolically challenging conditions, but not to meal timing per se. For instance, calorie restriction modifies the phase of the SCN clock and augments the phase shifting effects of light (88). Moreover, hypercaloric (ie, high-fat) feeding affects the SCN clock by lengthening its period and reducing its sensitivity to phase-shifting light exposure (89, 90).

Under normal conditions, the temporal organization of locomotor activity is controlled by the master clock. However, enforced locomotor activity during the usual rest period can have synchronizing effects on the SCN, thus providing feedback cues to the pacemaker. Behavioral activation in nocturnal rodents, such as novelty-induced wheel-running, transient hyperactivity, and/or stimulated arousal during the usual rest period all produce significant phase advances of the SCN clock (91–93). In humans, exercise of high intensity at the beginning of the night (ie, the early rest period) and at the beginning of the subjective day (ie, early wake time) induces phase delays and phase advances of the onset of melatonin secretion, respectively, used here as a marker of the phase of the master clock (94). In contrast to the SCN, peripheral clocks such as those in the pituitary gland can also be sensitive to the synchronizing effects of body temperature cues (95, 96).

### D. Internal hormonal and non-hormonal synchronizers

One major route by which the master clock has been demonstrated to distribute circadian signals to peripheral clocks is via blood-borne signals (97, 98). Evidence has accumulated for a key role of two endocrine rhythms tightly controlled by the master clock: GCs and melatonin.

Rhythmic secretion of adrenal GCs provides temporal cues to numerous target tissues expressing GR. The role of the circadian rhythm of circulating GC levels as internal zeitgeber has been demonstrated for many peripheral organs and brain regions. The functional significance of this rhythm for the circadian system is the central theme of this review, and a detailed description of the experimental evidence is given in *Section IV*.

Melatonin is synthesized by the pineal gland and secreted exclusively at night (in both nocturnal and diurnal species) under tight control by the master clock via a multisynaptic pathway. The daily rhythm of plasma melatonin distributes temporal cues to a multitude of target tissues expressing melatonin receptors (99). For example, in the pars tuberalis of the adenopituitary, rhythmic oscillations of clock genes (ie, *Cry1* and *Per1)* are under the control of circulating melatonin (100). Studies on pinealectomized rats indicate that endogenous melatonin acts as an internal zeitgeber, enhancing synchrony within the circadian system (101). The presence of melatonin receptors in the SCN shows that endogenous melatonin may have feedback effects on the master clock. During embryonic development, maternal melatonin provides time cues to the SCN and adrenal glands of the fetus (102, 103). In adults, pharmacological doses of melatonin administered in the late (subjective) day produce phase advances of the master clock in both nocturnal and diurnal species, including humans (99).

Another route by which the master clock distributes circadian signals to peripheral organs is through the projections of the sympathetic and parasympathetic branches of the autonomic nervous system. The innervation of the liver has attracted much attention in that regard (104). In particular, treatment with the sympathetic neurotransmitter noradrenaline up-regulates transcription of *Per1* mRNA levels in the liver both in vivo and in vitro. Moreover, electrical stimulation of the sympathetic nerves or adrenaline injection triggers *Perl* transcription measured by bioluminescence in the liver of transgenic *Per1-luc* mice (105). Similarly, in the adrenal gland, autonomic innervation serves as a time signal to adrenal endocrine rhythms (106, 107). By the same route, light exposure directly activates adrenal *Per* gene expression independently of the SCN clock (108, 109). Other organs with well-studied autonomic control by the SCN include the pineal and sub-maxillary glands (106, 107, 110, 111).

## III. The Circadian Rhythmicity of Glucocorticoid Release

### A. Normal circadian and pulsatile variations of glucocorticoid release

GC secretion is characterized by both circadian and 60- to 90-minute (ie, ultradian) oscillations. As detailed below, outputs from the SCN activate rhythmic release of corticotropin-releasing hormone (CRH) that stimulates circadian release of corticotropin (ACTH). The circadian rhythm of adrenal GC secretion is primarily dependent on this circadian pattern of ACTH release. In addition, neuronal signals generated by the SCN are transmitted by a multi-synaptic neural pathway to the adrenal cortex (106). In the adrenal cortex, an intrinsic circadian oscillator gates the physiological adrenal response to ACTH, defining a time window during which the adrenals most effectively respond to ACTH (22, 112).

**Figure 5.**
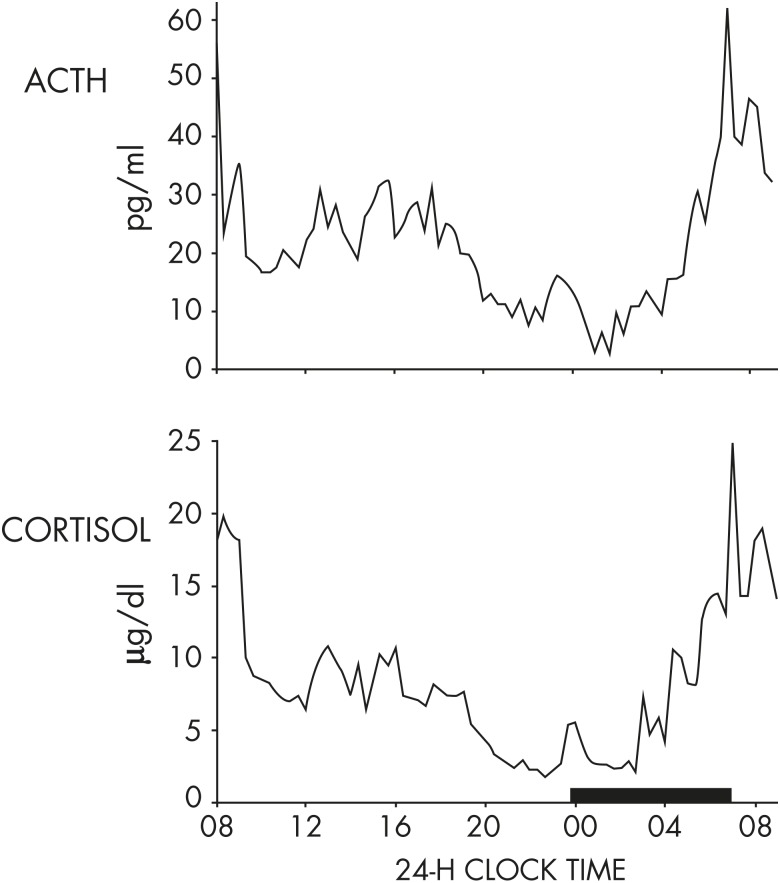
Representative 24-hour profiles of plasma ACTH and cortisol levels sampled at 15-minute intervals in a healthy young man studied under normal conditions. During scheduled sleep times (shown as a black bar), the sampling catheter was connected to plastic tubing extending into the adjacent room to avoid disturbing the subject. The parallelism of both pulsatile and circadian variations of ACTH and cortisol concentrations is evident.

In humans, under normal conditions, the 24-hour profiles of ACTH and cortisol show an early morning maximum, declining levels throughout daytime, a quiescent period of minimal secretory activity usually centered around midnight, and an abrupt elevation during late sleep culminating in an early morning maximum (113). An example of simultaneous profiles of ACTH and cortisol in a healthy young man studied under normal conditions is shown in Figure 5. In nocturnal rodents, the levels of corticosterone peak around the light-dark transition when the animals become active (107).

**Figure 6.**
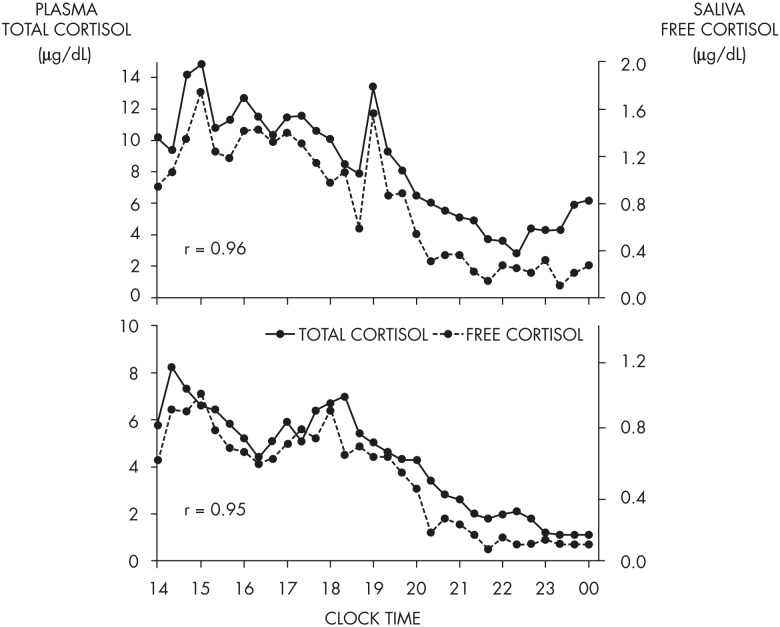
Simultaneous profiles of plasma total cortisol (solid line) and saliva free cortisol (dashed line) in two healthy young adults who were each submitted to blood sampling via an indwelling catheter at 20-minute intervals from 9 AM to midnight and provided a saliva sample at the time of each blood sampling. Caloric intake was exclusively in the form of a glucose infusion at a constant rate of 5 g/kg/24 h. Simultaneous levels of plasma cortisol and saliva cortisol were highly correlated in both individuals (r: Pearson correlation coefficient). [Unpublished illustration of data included in A. Guyon et al: Adverse effects of two nights of sleep restriction on the hypothalamic-pituitary-adrenal axis in healthy men. *J Clin Endocrinol Metab.* 2014;99(8):2861–2868 (117), with permission. © The Endocrine Society.]

Around the time of the nadir of HPA axis activity, concentrations of ACTH and cortisol in humans can be near the limit of detection of routinely used assays. Morning maxima of cortisol concentrations are often within the range of pathological values for afternoon and evening levels. Overall, the amplitude of the circadian variation of plasma cortisol is among the largest that can be detected in the human endocrine system (114). There is substantial evidence from studies in nocturnal rodents that the amplitude of the ACTH rhythm is considerably lower than that of the corticosterone rhythm, indicating that mechanisms downstream of pituitary control enhance the circadian variation of GC production. In humans, as evident in the example shown in Figure 5, the amplitude of the ACTH rhythm is more similar to that of the cortisol rhythm. Under normal conditions, the acrophase of pituitary-adrenal periodicity occurs between 6 and 10 AM and the nadir between 10 PM and 2 AM. Mathematical derivations of secretory rates from plasma concentrations have suggested that the 24-hour profile of plasma cortisol reflects a succession of secretory pulses of magnitude modulated by a circadian rhythm with no evidence of tonic secretion (114). With a 15-minute sampling interval, 12 to 18 significant pulses of plasma ACTH and cortisol per 24-hour span can be detected (115). Circadian and pulsatile variations parallel to that of cortisol have been demonstrated for the plasma levels of several other adrenal steroids, in particular, dehydro-epiandrosterone (116, 117). Circadian variations in GC release can also be detected in urine and feces with phase delays compared to blood (118–120). As illustrated in Figure 6, levels of free cortisol can be reliably measured in saliva and are highly correlated with circulating concentrations of total cortisol under normal conditions (117).

### B. Control of the circadian variation of glucocorticoid levels

**Figure 7.**
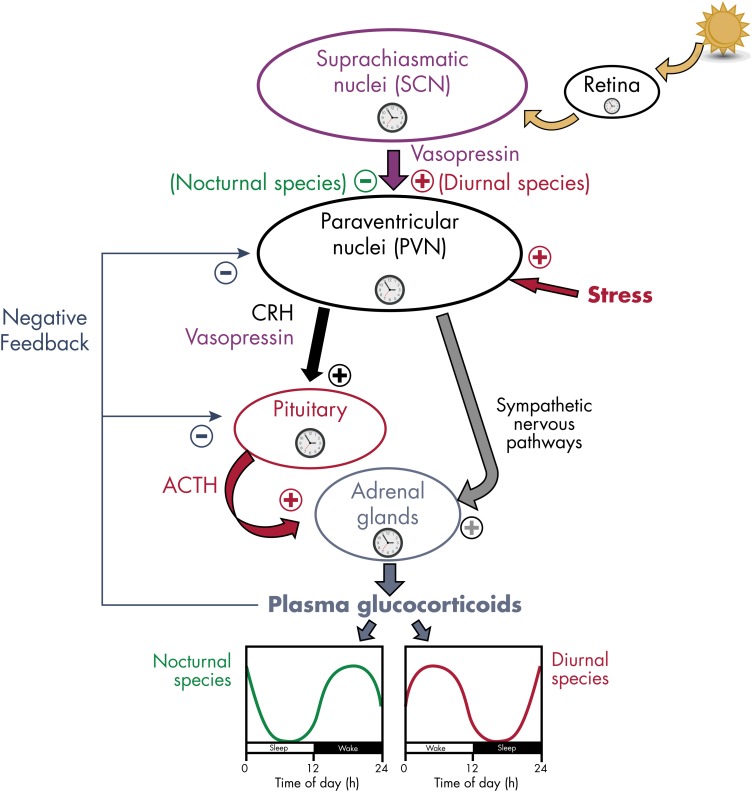
Schematic representation of the control of the circadian rhythmicity of GC release in mammals. Most components of the HPA axis contain circadian oscillators. The circadian secretion of GC is dependent on the rhythmic release of ACTH and a gating process by the adrenal clock. GC secretion is also modulated by nervous signals coming from the PVN of the hypothalamus via sympathetic nervous pathways. ACTH release is controlled by the rhythmic release of CRH and vasopressin from the PVN. Rhythmic activity of the HPA axis is under the control of the master clock in the SCN, reset by ambient light via the retina. The peak of vasopressin release from the SCN to the PVN region occurs during daytime in both nocturnal and diurnal rodents. In nocturnal rats, vasopressin exerts an inhibitory action on the PVN (probably via activation of *γ*-aminobutyric acid-containing interneurons), thus reducing GC secretion during daytime (green curve). By contrast, in diurnal grass rats, vasopressin stimulates PVN activity (probably via activation of glutamatergic interneurons), thus increasing GC secretion during daytime (red curve). Clock symbols represent self-sustained oscillators.

Circadian variations in GC release are governed by a complex interaction of central and peripheral clocks in the SCN, the PVN of the hypothalamus, the pituitary corticotrophs, and the adrenocortical cells producing steroids (121). Figure 7 provides a schematic representation. The pivotal role of the SCN in the circadian control of GC release was recognized in 1979, when destruction of the SCN in rats was shown to eliminate the rhythms of ACTH and corticosterone (122). The importance of neural connections with other hypothalamic regions was recognized when SCN grafts in animals with complete ablation of the SCN was found to restore rhythms of locomotor activity, but not of circulating corticosterone (123).

Control by the master clock in the SCN is mediated via two pathways, a neuroendocrine path (ie, the HPA axis) and a nervous path (ie, the sympathetic system) (31, 107). In rats, SCN pacemaker neurons rhythmically release the neuropeptide vasopressin (124), which inhibits CRH neurons in the PVN, the structure that constitutes the top of the HPA axis. The fact that the SCN control of the HPA axis rhythm involves inhibitory mechanisms was first suggested by the fact that SCN lesions resulted in corticosterone increases at the time of the trough of the rhythm. Vasopressin release from the SCN is high during the light phase, and interestingly, PVN neurons of diurnal species as compared to nocturnal species respond differently to vasopressin stimulation (107, 125). This control of CRH activity in the PVN may occur via vasopressin release into the cerebrospinal fluid or extracellular space (64), via direct projections from the SCN to the PVN (126), or via indirect projections to subparaventricular GABAergic interneurons that in turn project to the PVN (125). It is likely that vasopressin is not the only SCN signal controlling the daily corticosterone rhythm. Overall, the rhythm seems to be controlled by a balance between inhibitory and stimulatory inputs from the SCN (107). The clockwork machinery is present in the PVN and has been shown to generate self-sustained oscillations of *Per1* expression when isolated in vitro (127).

Although experimental evidence is scarce, the PVN clock might be involved in the 24-hour variation of CRH mRNA levels that peaks at night in rats (128, 129). The negative feedback of GCs controls the release of pituitary ACTH, with higher sensitivity during the nadir (trough) of the 24-hour rhythm. A role for vasopressin release from the PVN in the control of rhythmic ACTH release has been suggested by human experiments where negative feedback regulation by GCs was eliminated by metyrapone treatment and CRH was infused iv at a constant rate. Under these conditions, a clear 24-hour rhythm of ACTH was observed, but it was set at a higher mean level due to the absence of negative feedback (130). High-affinity MRs in limbic structures, such as the hippocampus, mediate the effect of GCs on the maintenance of basal activity in the HPA axis at that nadir time (late evening in humans, early morning in nocturnal rodents). The more abundant GRs in the hippocampus are thought to play an important role when the HPA axis is activated (at the time of the circadian peak and during stress responses; see *Box 2*) (131). More recently, the MR, bound to the plasma membrane, has been shown to be involved in the initial steps of the response to psychological stress (132) (see *Box 1*).

After secretion of CRH in the portal system of the median eminence, ACTH synthesized in the anterior pituitary is released into the circulation and stimulates GC secretion from the adrenal cortex. In rats, *Pomc* mRNA levels in the anterior pituitary do not show significant 24-hour rhythms despite the fact that clock genes appear to be expressed and to oscillate in anterior pituitaries from both rodents and humans (52, 129, 133, 134). Therefore, *Pomc* transcription is probably not a clock-controlled process in the pituitary. Plasma ACTH levels in rats are higher in the evening compared to morning, and this dusk increase in ACTH is dampened by SCN lesions (135, 136). Further findings indicate that the SCN increases adrenal sensitivity to ACTH, especially during the rising portion of the 24-hour rhythm of corticosteroids. This modulation of the sensitivity of the adrenal glands to the incoming ACTH message is partly mediated through autonomic pathways (106, 137, 138). More precisely, SCN and other signals integrated in the PVN are transmitted to neurons in the intermediolateral column of the spinal cord, which then project via splanchnic fibers directly to the adrenal glands (106, 138).

Although little is known about circadian modulation at the level of the PVN or the pituitary, work with transgenic mice suggests that local clocks in adrenocortical cells gate the sensitivity of the adrenal steroidogenic pathway to ACTH stimulation (22, 139, 140). Adrenal slice cultures from wild-type animals show different responses to ACTH stimulation depending on the time of treatment (22, 141). Adrenal slices from animals with genetically disrupted clock function lack this capability (22). Furthermore, adrenal function and responses to both acute and chronic stress are altered in mice with defective circadian clocks due to genetic ablation of *Bmal1* (142). Together, these data suggest that a stable phase-relationship between central (SCN) and peripheral (adrenocortical) clocks is necessary to maintain robust high-amplitude circadian rhythms of GC release (143).

### C. The 24-hour profile of glucocorticoid secretion is a robust marker of the central circadian signal

Consistent with its role as the main hormonal signal synchronizing the master circadian pacemaker with a multitude of peripheral clocks, the 24-hour cortisol profile is relatively unaffected by acute changes in environmental and behavioral conditions.

**Figure 8.**
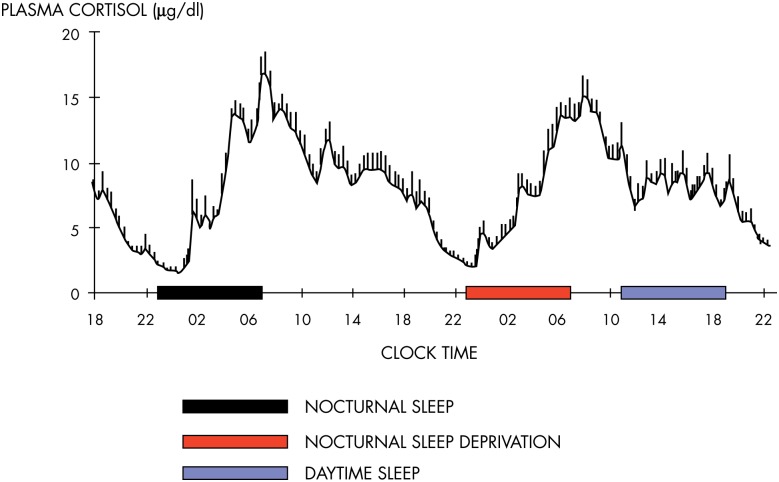
Mean profiles of plasma cortisol concentrations measured at 20-minute intervals from eight healthy young men studied over a 53-hour period including a night of nocturnal sleep (black bar), 28 hours of continuous wakefulness in a semirecumbent position with a night of total sleep deprivation (red bar), and an 8-hour period of daytime recovery sleep (blue bar). Caloric intake was exclusively in the form of an iv glucose infusion at a constant rate. The persistence of the circadian rhythmicity of plasma cortisol despite these drastic manipulations of the light-dark cycle, sleep-wake cycle, and feeding schedule is evident. Note that the absence of wake-sleep and sleep-wake transitions during the night of total sleep deprivation results in a slight dampening of the amplitude of the circadian variation. [Redrawn from E. Van Cauter et al: Modulation of glucose regulation and insulin secretion by circadian rhythmicity and sleep. *J Clin Invest.* 1991;88(3):934–942 (144), with permission. © American Society for Clinical Investigation.]

The mean profile of cortisol levels shown in Figure 8 illustrates the persistence of the cortisol rhythm after an acute shift of the light-dark cycle and sleep-wake cycle and in the absence of alternations between feeding and fasting. Eight normal young men were studied during a 53-hour period that included 8 hours of nocturnal sleep in total darkness, 28 hours of continuous wakefulness in a semirecumbent position with constant indoor light exposure, and 8 hours of daytime sleep (144). Caloric intake was replaced by an iv glucose infusion at a constant rate. It can be seen that the overall wave shape of the profile was only modestly affected by the absence of sleep or the presence of sleep at an abnormal time of day. During the 28 hours of constant recumbent posture, continuous wakefulness, constant exposure to indoor light, and constant caloric intake, the normal wave shape of the rhythm of GC release was clearly maintained. Similar findings have been obtained in multiple laboratories (145).

Adaptation of the 24-hour rhythm of cortisol to an abrupt phase shift of dark-light cycle, sleep-wake cycle, and feeding schedule, as occurs in jet lag and in certain types of shift work rotation, requires multiple days, usually 1 day per time zone in the case of jet lag. When the change in sleep-wake and dark-light cycle occurs gradually, the shift in the cortisol rhythm correlates with the shift in the rhythms of other processes known to be under central circadian control, including melatonin release, body temperature, and subjective alertness (32). An abundance of similar evidence linking the characteristics of outputs from the master clock with the rhythm of cortisol release has led to the widespread use of the 24-hour rhythm of plasma cortisol in humans and of corticosterone in rodents as a marker of central circadian timing.

**Figure 9.**
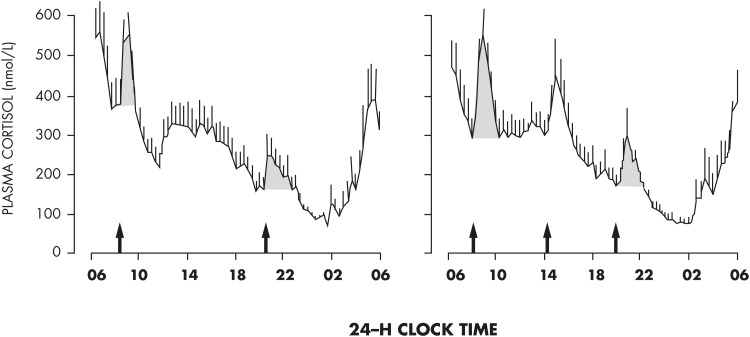
Mean profiles of plasma cortisol concentrations measured at 20-minute intervals from eight healthy young men who each participated in a randomized clinical trial comparing the impact of either two or three identical carbohydrate-rich meals presented at fixed clock times. A clear short-term increase in cortisol concentrations occurs after each meal but does not affect the overall wave shape of the circadian profile. Thus, acute changes in feeding schedules that have a major effect on peripheral circadian oscillators in metabolic tissues do not readily desynchronize the GC rhythm from the central circadian pacemaker, consistent with its role as a robust internal synchronizing signal. [Adapted from E. Van Cauter et al: Circadian modulation of glucose and insulin responses to meals: relationship to cortisol rhythm. *Am J Physiol.* 1992;262(4 Pt 1):E467–E475 (146), with permission. © American Physiological Society.]

However, the overt rhythm of GC release can be affected by multiple stimuli that evoke transient stimulatory or inhibitory responses. For example, modulatory effects of sleep-wake transitions are well recognized and are discussed in *Section V*. Feeding and fasting have potent effects on GC secretion and can therefore also affect the shape of the 24-hour profile of GC levels. In humans, under normal conditions, a short-term elevation of cortisol levels occurs reliably after ingestion of a carbohydraterich meal presented at 12- or 6-hour intervals (Figure 9). The detection of this meal response requires a strict standardization of the timing and composition of the meal for a group of subjects in order to be differentiated from spontaneous pulsatile release. The profiles shown in Figure 9 indicate that these evoked meal responses do not affect the overall amplitude and phase of the circadian rhythm of cortisol release (146). Of note, in this study, the duration of fasting between meals was not long enough to cause a reduction of glucose levels approaching the hypoglycemic threshold that might have triggered a counter-regulatory increase in cortisol release. The first line of glucose counter-regulation is activated around 3.75 mmol/L (68 mg/dL) and involves increased release of glucagon, adrenaline, and GH (147). An increase in cortisol does not occur until glucose levels are below 3.5 mmol/L (63 mg/dL). In nocturnal rodents (in which intermeal fasting periods are habitually short), a nutritional challenge consisting of restricting daily food access to a few daytime hours (ie, during their resting period) results in the appearance of a food-anticipatory rise in circulating corticosterone before the mealtime, in addition to the nocturnal rise controlled by the master clock. The additional daytime peak is ACTH independent (148, 149) and has been associated with noradrenergic activation of the PVN (150). Concomitant with the anticipatory rise in corticosterone before mealtime, animals manifest bouts of motor activity and thermogenesis driven by a network of cerebral clocks outside the SCN (151). In humans, daily profiles of plasma cortisol have been studied during the traditional Muslim Ramadan, a 1-month period when caloric intake and water intake are restricted to the nighttime. In addition to the morning rise of plasma cortisol, a secondary increase in cortisol was observed in the late afternoon, before the expected time for eating (87, 152). These studies are limited by infrequent blood sampling at 4-hour intervals or longer, and because glucose levels were not measured, it remains unclear whether the 18-hour fast elicited counter-regulatory cortisol release.

Environmental lighting conditions matter not only for resetting the master clock, but also for modulating adrenal activity. In mice, light exposure at night triggers corticosterone release via sympathetic pathways without inducing ACTH release (108). Conflicting results have been obtained in rats in which light exposure at night leads to a decrease in blood corticosterone (106). Nevertheless, in both studies, the effect of light appears to be ACTH-independent and mediated by sympathetic inputs originating from the PVN. In humans, bright light in the early morning, but not in the afternoon, induces a robust transient elevation of plasma cortisol, highlighting the strong modulatory effect of light conditions on the rhythmicity of the human HPA axis (153).

In rodents, chronic exercise increases the daily peak of plasma corticosterone. This occurs mainly by an increased synthesis of corticosteroid in the adrenals (154, 155). In humans, acute voluntary exercise also produces a transient stimulation of cortisol secretion (156, 157).

Exposure to stressors can acutely increase ACTH and GC release, but the response is generally superimposed on the 24-hour endogenous rhythm, without intrinsic effect on either phase or amplitude. The HPA axis shows daily variations in its responsiveness to acute exposure to various stressors. In conscious rats, corticosterone induction as a marker of HPA reactivity in response to noise, restraint stress, and ACTH administration is larger in the early light phase when spontaneous corticosterone activity is low compared to the early dark phase when spontaneous activity is high (136, 137, 158). The HPA response to insulin-induced hypoglycemia is also time-of-day dependent, but with opposite direction compared to psychological stressors (eg, restraint) (159). However, in chronically stressed humans, cortisol levels are often elevated throughout the day, thus blunting the normal day-night variation (160, 161). Well-documented examples include major depressive disorder (162–164) and chronic insomnia (165).

### D. Contrasting diurnal and nocturnal species

The 24-hour rhythm of corticosteroids is oppositely phased between diurnal and nocturnal mammals with respect to the light-dark cycle (ie, dawn and dusk, respectively; see Figure 7), but it anticipates the onset of the daily period of wakefulness and activity in both groups (158, 166), participating in the mobilization of energy stores and the stimulation of appetite (167).

In contrast, molecular oscillations of the master clock and its outputs (eg, vasopressin) are phase-locked with respect to the light-dark cycle in both day- and night-active species (63, 168, 169). Together, these data suggest that the functional switch between diurnal and nocturnal behaviors occurs downstream of the SCN (probably in the first-order targets of SCN output signals) (170). In keeping with that hypothesis, intracerebral injections of vasopressin have opposite effects on HPA axis activity between nocturnal laboratory rats and diurnal Sudanian grass rats, with respectively inhibitory and stimulatory actions on corticosterone release (125) (see Figure 7). These findings suggest that the neurochemical nature of the subparaventricular interneurons mediating these effects (ie, GABAergic or glutamatergic, respectively) may participate to the diurnal vs nocturnal niche of the animal.

The opposite phasing of GC rhythms and sleep-wake cycles relative to SCN neuronal activity, melatonin release, and the light-dark cycle between diurnal and nocturnal species has raised concerns about the suitability of nocturnal rodents as model systems for developing drugs that target the human HPA axis and metabolism. Further studies in diurnal mammalian species, such as Sudanian grass rats, dogs, or rhesus monkeys, may thus provide important insights into the clinical significance and pathophysiological mechanisms underlying conditions of abnormal GC rhythmicity and help in the design of therapeutical approaches.

### E. Control and functional significance of glucocorticoid pulsatility

In every mammal studied so far, GCs are released in a pulsatile fashion with large amplitude pulses during the active awake period and infrequent low amplitude pulses during the early hours of sleep. This pulsatile activity emerges as a natural consequence of the feed-forward feedback relationship between the anterior pituitary corticotrophs and the zona fasciculata of the adrenal cortex (171). Indeed, infusion of constant levels of CRH during the nadir of HPA activity is sufficient to result in pulsatile ACTH and corticosterone secretion (172), whereas blocking the activity of the SCN abolishes circadian rhythmicity but allows ultradian rhythmicity to continue throughout the 24 hours (173).

Pulsatile ACTH and GC secretion results in an oscillating signal of hormone ligand, and therefore it is likely that the receptor signaling pathways have adapted to decode this oscillatory signal. This appears to be the case both for the adrenal cortex, which preferentially responds to pulsatile signals of ACTH, and for other tissues expressing GR around the body (174, 175). Because GCs secreted by the adrenal cortex are synthesized de novo, the pulsatile activity of this gland must be the result of a very dynamic and tightly controlled process. Recent evidence suggests that this activity is regulated via a fine balance between ACTH-mediated feedforward and GC-mediated intra-adrenal feedback (176). These pathways are activated by protein kinase A-mediated genomic and nongenomic mechanisms (177). Interestingly, in critical illness, although pulsatility is maintained, there appears to be a change in intra-adrenal signaling, resulting in a markedly increased sensitivity to circulating ACTH (178).

We now know that there is a differential response of many genes to constant vs pulsatile administration of GCs (179), and in vivo studies have confirmed that each individual pulse of GC is associated with cyclical binding of the GR and coregulatory molecules to promoter sequences of GC response genes and cyclical activation of transcription (180). Recent evidence suggests that the differential responses to different temporal patterns of GC ligand depend on spatiotemporal chromatin dynamics reflecting transient activation of distal regulatory elements (181).

The differential effects of pulsatile vs constant GC are not only seen at the molecular level. When corticosterone replacement is given to adrenalectomized rats, animals given pulsatile replacement therapy have a normal neuroendocrine and behavioral response to a 10-minute noise stress, whereas animals given exactly the same dose of GC, but as a constant infusion, have dulled responses (182). Interestingly, these animals also show a stress-induced neuronal activation in the amygdala that is dependent upon the pulsatility of the infused corticosterone (182). Recent work reveals that the ultradian pulsatile pattern plays an important role for normal glutamatergic synaptic transmission (183).

These animal studies clearly show that for normal transcriptional and behavioral responses, it is necessary that GCs are presented to their target tissue in a pulsatile fashion rather than in a smooth pattern. The question arises, of course, as to how important this may be in humans. To date, there have been relatively few studies of the importance of cortisol pulsatility in human pathophysiology, although there is evidence for altered pulse dynamics in depression (184) and in obstructive sleep apnea (185).

## IV. Impact of the 24-Hour Rhythm of Glucocorticoid Levels on Central and Peripheral Oscillators

The 24-hour rhythm of adrenal corticosteroids provides temporal cues to circadian clocks and physiological rhythms in numerous central and peripheral tissues expressing GR. Conversely, disturbances of the physiological rhythm of GCs by chronic continuous administration of synthetic corticosteroids such as prednisolone or by adrenalectomy have been experimentally shown to markedly alter circadian oscillations of clock gene expression in multiple tissues (186). It is important to note that negative findings regarding the impact of adrenalectomy (a condition where GC concentrations are at nadir levels throughout the 24-hour cycle) on a given clock system do not exclude the possibility that an acute elevation of GC concentrations at the normal nadir time in an adrenalectomized animal will not disrupt rhythmic clock expression in target tissues. Multiple studies have obtained evidence for a role of GC in synchronizing local clocks by demonstrating altered rates of re-entrainment in adrenalectomized animals (187).

### A. Synchronization of non-SCN brain clocks and rhythms

Table 1 lists the central nervous system regions for which there is evidence of rhythmic expression of clock genes as well as expression of GR, and it summarizes the evidence for a potential implication of GCs in regulating local oscillators and rhythms. All the findings derive from rodent studies.

**Table 1.** **Evidence for GR Regulation of Local Circadian Clocks and Rhythms in Brain Tissues**

Tissue	Evidence for Presence of Local Clocks	Expression of GRs	Do GCs Impact Local Clocks or Rhythms?	Potential Functional Significance of Local Clock and Impact of GC	Selected Refs. Investigating the Impact of GCs on Cellular Rhythms
SCN	Yes	No	No	Robustness of the master circadian signal relative to disturbances of GC levels or rhythmicity	200
Pineal	Yes	No	No	Robustness of the other hormonal internal zeitgeber relative to disturbances of GC levels or rhythmicity	205
PVN	Yes	Yes	Yes	Modulation of stress response and sympathetic tone	189, 192
Arcuate nucleus	Yes	Yes	Unknown	Modulation of hunger, appetite, and feeding	N/A
Central amygdala	Yes	Yes	Yes	Modulation of emotion	196
Bed nucleus of stria terminalis	Yes	Yes	Yes	Modulation of reward, anxiety, and stress	196
Raphe nucleus	No	Yes	Yes	Modulation of feeding, arousal, mood, sleep, cognition, and master clock	199
Hippocampus	Yes	Yes	Yes	Modulation of stress, emotion, memory, neurogenesis, response to antidepressant treatment	197, 198

Abbreviation: N/A, not available.

#### 1. Suprachiasmatic nuclei

The master SCN clock does not express sizeable amounts of GR, except during the first postnatal week in rats (188). Adrenalectomy has no major effect on rhythms of circadian gene expression in the SCN (189). GCs may, however, influence the central circadian signal in an indirect fashion via behavioral modulation, for example, during re-entrainment of activity rhythms after jet lag (190).

#### 2. Paraventricular nucleus

Adrenalectomy in rats reduces the amplitude of the daily expression of *Per1*, suggesting that the daily rhythm in corticosterone modulates robustness of the circadian rhythmicity in the PVN (189). Furthermore, acute stress induces expression of the clock gene *Per1* in the PVN (191, 192).

#### 3. Arcuate nucleus

The arcuate region displays self-sustained in vitro oscillations, as assessed with *PER2::LUC* mice (193), and GRs are expressed in this area (194, 195). To our knowledge, the possible effect of GCs on the arcuate clock has not yet been investigated.

#### 4. Limbic areas

Several limbic regions (eg, central amygdala and bed nucleus of the stria terminalis) show daily oscillations of clock proteins that are suppressed by adrenalectomy. In these adrenalectomized animals, rhythmic expression of the clock protein PER2 can be restored by exposure to a daily rhythm of corticosterone levels obtained by exogenous administration in the drinking water (196). Expression of the clock gene *Per1* in the hippocampus is also modulated by circulating GCs (197, 198).

#### 5. Raphe

GC-mediated resetting of physiological rhythms has also been observed in the raphe nuclei, although this brain region does not express clock genes. In this brain region, circulating corticosterone levels indeed control the rhythmic transcription of tryptophan-hydroxylase, the ratelimiting enzyme of serotonin synthesis (199).

In summary, there is substantial evidence that the 24-hour rhythm of plasma GCs normally generated by the adrenal glands participates in the adaptation of daily rhythmicity in multiple brain tissues.

### B. Synchronization of peripheral clocks and rhythms

As listed in Table 2, rhythmic expression of circadian genes has been detected in a wide variety of peripheral tissues that also express GR. Furthermore, in almost all of the tissues examined so far, an impact of modulation of GC levels and/or rhythmicity on the peripheral clock has been demonstrated. Except where indicated below, the findings derive from rodent studies.

**Table 2.** **Evidence for GR Regulation of Local Circadian Clocks and Rhythms in Peripheral Tissues**

Tissue	Evidence for Presence of Local Clocks	Expression of GRs	Do GCs Impact Local Clocks or Rhythms ?	Potential Functional Significance of Local Clock and Impact of GC	Selected Refs. Investigating the Impact of GCs on Cellular Rhythms
Liver	Yes	Yes	Yes	Modulation of gluconeogenesis, lipid metabolism	200, 203
Muscle	Yes	Yes	Yes	Modulation of muscle atrophy, sarcopenia	186, 207
Pancreatic *β*-cells	Yes	Yes	Unknown	Modulation of glucose tolerance, diabetes risk	N/A
White adipose tissue	Yes	Yes	Yes	Modulation of lipid metabolism, appetite regulation, inflammation	189, 211
Gut	Yes	Yes	Unknown	Altered intestinal lipid absorption	N/A
Heart	Yes	Yes	Yes	Modulation of atrioventricular transmission	200
Kidney	Yes	Yes	Yes	Modulation of filtration, volume regulation	200, 205
Lung	Yes	Yes	Yes	Modulation of immune function	218, 219
Skin	Yes	Yes	Yes	Modulation of immune function, hair cycle	200, 221
Ovary	Yes	Yes	Yes	Modulation of steroidogenesis, follicle survival	222
Blood (mononuclear cells)	Yes	Yes	Yes	Immune function	219, 226, 227
Salivary gland	Yes	Yes	Yes	Unknown	205
Bone	Yes	Yes	Yes	Modulation of osteoclast survival, bone resorption	230
Cornea	Yes	Yes	Yes	Modulation of local immune function, lymphangiogenesis	205
Pituitary gland	Yes	Yes	Yes	Unknown	205

Abbreviation: N/A, not available. Except where indicated in the main body of the text, the findings derive from rodent studies.

#### 1. Liver

Treatment with dexamethasone, a potent synthetic GC, is a potent synchronizer of the liver clock in mice, with the direction and amplitude of the phase depending on the timing of treatment (200, 201). In vitro treatment with prednisolone also affects clock gene expression in cultured hepatocytes, whereas continuous administration of this synthetic GC in mice attenuates the daily oscillations of clock genes in the liver (186). Lesions of the SCN lead to arrhythmicity of the liver transcriptome, whereas a single injection with dexamethasone restores daily rhythms in hepatic gene expression (202). Adrenalectomy in mice affects the circadian expression of metabolic genes, such as glucokinase or 3-hydroxy-3-methyl-glutaryl-coenzymeA reductase, but not that of clock genes (203, 204). However, in vitro circadian oscillations of livers sampled from adrenalectomized rats are delayed compared to sham-operated control animals (205). Chronic mild stress disturbs daily patterns of hepatic expression in metabolic and clock genes (206).

#### 2. Kidney

Dexamethasone or corticosterone shifts clock gene expression in that tissue (200, 201), whereas adrenalectomy delays the phase of ex vivo *Per1* oscillations (205).

#### 3. Skeletal muscle

Circadian expression of genes in rat skeletal muscles can be modulated by corticosterone (207), whereas continuous treatment with prednisolone diminishes the daily amplitude of clock gene expression in the mouse muscle (186).

#### 4. Pancreatic β-cells

Marcheva et al (208) demonstrated the existence of robust autonomous circadian oscillations of *Per2,* associated with rhythmic expression of genes involved in insulin signaling, glucose uptake and metabolism, and *β*-cell growth and proliferation. Animals in which the circadian clock in the pancreas has been genetically manipulated have defects of insulin release and abnormal glucose homeostasis (208, 209). In healthy humans under conditions of constant glucose infusion, insulin secretion rates show a circadian variation that is inversely related to the cortisol rhythm, with a significant correlation in the amplitudes of the two rhythms (144).

#### 5. White adipose tissue

Dexamethasone increases and attenuates the amplitude of clock gene oscillations in preadipocytes and differentiated adipocytes, respectively (210). Furthermore, adrenalectomy in rats suppresses oscillations of metabolic genes, such as *leptin, adiponectin,* or *peroxisome proliferator-activated receptor α,* and diminishes the amplitude of clock gene oscillations, including *Per1* and *Rev-erb α* (189). In human adipose explants, dexamethasone treatment alters clock gene expression (211).

#### 6. Gut

The expression of various clock genes has been documented in parts of the intestine (212). The colon exhibits the highest expression of clock genes (213, 214). The expression of clock genes increases from duodenum to colon and from mucosal cells to epithelial cells. Clock genes in the jejunum and colon show circadian variations (212–214) that are in phase with their rhythmic expression in the liver but are phase delayed compared to their temporal expression in the SCN (75). It has recently been shown in mice that the intestinal microbiota exhibit endogenous circadian rhythmicity that is partly synchronized by host feeding and dependent on diet composition (215–217). Reciprocally, circadian variations of gut microbes affect rhythms of clock gene expression in the mediobasal hypothalamus and in the liver. High-fat diet markedly alters circadian patterns of gut microbial structure and function, resulting in the disruption of both central and liver clocks with adverse metabolic consequences. It is not known whether circadian variation in GCs may play a role in the interactions between rhythms in the microbiota and rhythms in the host.

#### 7. Lung

The epithelial cells of the lung express clock genes that are sensitive to activation of GC signaling (218). Daily injections of corticosterone modify expression patterns of clock genes in the rat lung (201), whereas the disappearance of rhythmic GCs is without d**e**tectable effects on the phase of *Per1* oscillations (205). In human bronchial epithelial cells, dexamethasone leads to an increased expression of the clock gene *Per1* (219).

#### 8. Heart

Dexamethasone is able to shift clock gene expression in the cardiac muscle (200, 220).

#### 9. Pituitary

Adrenalectomy hardly affects the daily expression patterns of clock genes in the hypophysis (133, 205) and does not affect the rate of re-entrainment after a delay or advance of the light-dark cycle (205). However, hydrocortisone treatment of adrenalectomized animals advanced the rhythm of *Per1-luc* expression.

#### 10. Salivary gland

There is no evidence for a role of the GC rhythm in synchronizing the clock in the salivary gland (205), indicating that other internal pathways are more important.

#### 11. Cornea

In rats, adrenalectomy delays *Per1-luc* oscillation of the cornea, this phase shift being partially counteracted by hydrocortisone treatment (205).

#### 12. Skin

Dexamethasone triggers expression of clock genes in cultured rat-1 fibroblasts (200). Clock gene oscillations in primary fibroblasts, keratinocytes, and melanocytes from human skin explants can also be phase-adjusted by dexa-methasone (221).

#### 13. Ovary

Cultured granulosa cells from ovaries of *Per2-luc* rats can be synchronized by dexamethasone treatment (222).

#### 14. Blood mononuclear cells

Clock genes are rhythmically expressed in blood nucleated cells (223, 224), whereas GR acetylation in these cells shows day-night variations (225). Timed treatment with GCs synchronizes the local clock in these cells in in vitro and in vivo conditions (226, 227).

#### 15. Bone

Metabolism of bone is modulated by clock genes (228). In turn, the bone clock in humans and mice can be reset by dexamethasone (229, 230).

### C. Impact on photic entrainment

It is worth mentioning that GCs can also modulate the entrainment of the SCN pacemaker to light. Notably, when animals are exposed to an abrupt shift in the light-dark cycle (jet-lag paradigm), blocking the synthesis of adrenal corticosteroids or submitting the animal to adrenalectomy changes the speed of re-entrainment of the circadian system to the new light-dark schedule (187, 190, 205). As mentioned earlier, the fact that GR are not found in the adult SCN suggests that these effects are mediated indirectly.

### D. Impact on entrainment by food intake

When combined with timed meal access, GC signaling can provide conflicting resetting cues, thus impairing entrainment of peripheral organs (204, 231). This modulatory effect may be tissue-dependent because the liver clock appears to be more sensitive to feeding cues, whereas the lung and kidney are more easily reset by corticosteroids (201).

### E. Reciprocal interaction: clock proteins modulate glucocorticoid receptor activity and bioavailability

Although there is ample evidence that GC rhythmicity provides synchronizing cues to a number of central and peripheral clocks (Tables 1 and 2), conversely, in a number of peripheral tissues including liver, white and brown adipose tissue, bone, prefrontal cortex, and aorta (232-235), the clock machinery has been shown to regulate GR transcription and activity (illustrated in the upper part of Figure 4). Via its acetyl-transferase activity, CLOCK can directly acetylate GRs, thereby reducing their transcriptional efficiency (236). Furthermore, CRY1 and CRY2 can repress GC transactivation (237). CHRONO has been shown to interact with both clock proteins and the GR (238). Together, these processes may produce a circadian gating of GC signaling in peripheral tissues, defining a temporal window of sensitivity for GC target gene regulation (239). Collectively, the functional significance of these findings is that optimal physiological effects of GCs occur when the central signal that controls the rhythm of GC release and the peripheral rhythms in tissues expressing GR are aligned.

The actions of GCs within target cells are determined not only by circulating concentrations and cellular receptor density, but also by the intracellular conversion of biologically inactive cortisone to cortisol by the enzyme 11 *β*-hydroxysteroid dehydrogenase type 1 (HSD-1) and by the rate of the reverse reaction by the enzyme HSD-2 (see *Box 2*). In humans and rodents, HSD-1 involved in the regeneration of GCs is widely expressed throughout the brain, including in hypothalamic areas, and in many peripheral tissues, including liver and adipose tissue (37, 240). In some tissues, HSD-1 expression itself is under circadian control, suggesting that local clock function may further modulate GR-mediated actions by regulating the tissue availability of the active hormone (241-243). The distribution of HSD-1 supports a role for this enzyme in the regulation of metabolism, appetite, and circadian rhythms (244).

## V. Impact of Circadian Rhythmicity on the Physiological Effects of Glucocorticoids

The multiple physiological actions of GCs are best described as “chronophysiological” because they are generally strongly modulated by circadian time. In the sections below, we briefly review the impact of the 24-hour rhythm of GCs on the stress response, metabolism, cardiovascular function, immune function, and sleep-wake regulation, as well as memory and learning processes.

### A. The stress response

A commonly used definition of a “stressor” is any stimulus that disturbs or threatens to disturb the homeostasis and integrity of the individual. The stress response is the spectrum of physiological and behavioral adaptations required to restore homeostasis (*Box 1*)*.* Table 3 lists the different domains involved in this multiplicity, and the list is by no means exhaustive. As remarked by Koolhaas et al (11), uncontrollability of exposure to a stressor or failure to cope is the most severe stressful condition. GC action as part of the stress response also displays an impressive diversity because it is cell-specific and context-dependent in coordinating the individual response to the challenge (245).

**Table 3.** **The Multidimensional Nature of the So-Called “Stress Response”: Diversity and Multiplicity**

Stressors	Stress Responses
Main target of exposure to stressor	Physical function, cognitive function, emotional regulation, social integration, development, maturation
Duration of exposure to stressor	Acute, single, repeated, prolonged, chronic
Severity of stressor	Mild, moderate, severe, life threatening
Timing of exposure to stressor	Predictable, unpredictable, dependent on biological time of day, early life, adult life, late life
Type of response to stressor	Homeostatic (adaptive, return to baseline set point); allostatic (maladaptive, variable set point)

Studies of the HPA response to a stressor have involved a wide variety of experimental challenges as well as life events. Examples of widely studied stressors and stress responses in humans include the cortisol awakening response (a partly predictable mild stressor) (246), high-intensity exercise (a physical stressor where the cortisol response facilitates metabolic adaptation but may be exaggerated by the psychological challenge) (247), different forms of mental arithmetic tasks performed under direct supervision with or without time pressure, and the Trier social stress test (TSST), a laboratory test where cognitive function is challenged in a socially adverse context (248). A chronic pathological condition that may arise from inadequate coping with terrifying and often violent situations is post-traumatic stress disorder (PTSD) (249). In animal models, a common experimental procedure to enhance stress responses includes subjecting pups to prolonged periods of maternal separation, an emotional and psychosocial condition that may lead to lifelong adverse health effects (250). During maternal absence, the pups become more responsive to common stressors. Acute stress paradigms include the forced swimming test (an acute exposure to a physical and psychological challenge that may be perceived as life-threatening), the restraint or immobilization test (an exposure to an aversive condition that is often repeated day after day for extended periods of time), and different forms of exposure to social defeat (a psychosocial stressor that is variable in its intensity depending on study conditions). Of note, there have been only a few studies examining the impact of circadian regulation on the HPA response to any of these stressors (136, 137, 158–160).

In the traditional conceptualization of stress as the “flight-or-fight” response, in a first line of defense against the external stressor, adrenaline and noradrenaline secreted from the adrenal medulla increase heart frequency and blood pressure, improve oxygen uptake in the lung, and stimulate release of glucose and fatty acids from body stores, thus fueling energy to organs involved in the flight-or-fight response, mainly muscles and the brain (251, 252). Release of cortisol requires de novo biosynthesis from cholesterol, thus lagging the autonomically controlled catecholamine secretion by several minutes. Cortisol frees additional energy by promoting liver gluconeogenesis (253) and inhibition of insulin production from the pancreas (254). Its vasoconstrictive action further improves blood circulation (255) and, thus, distribution of oxygen to muscles and the brain. Central effects include stimulation of cognitive function and alertness (256).

In rodent models, exposure to a psychosocial stressor can disrupt overt expression of behavioral circadian rhythms (257) and of the rhythm of circulating GC. At the molecular level, restraint stress and forced swimming trigger a transient up-regulation of transcription of the clock gene *Per1* in clocks of peripheral tissues and in the PVN of mice. This transcriptional induction occurs independently of the time of day, via activation of a GC-responsive element *(GRE)* in the *Perl* promoter region (191, 258). Activated GR can also increase *Per2* transcription, through interaction with BMAL1 (259) and via down-regulation of *Rev-erb α* (260).

When stress becomes chronic and cortisol levels stay elevated, particularly at the time of the usual nadir, some of the beneficial actions of cortisol become inverted. Evidence from both rodent and human studies indicates that chronic exposure to even modestly elevated cortisol concentrations can result in memory deficits, immunosuppression, central obesity, as well as cardiometabolic and reproductive disturbances (161, 261–267). To understand this switch from protective to damaging effects of cortisol, the specific roles of both MR and GR in the various stages of information processing in the brain should be taken into account. The effect of cortisol on cognition can turn from adaptive to maladaptive when actions via the two corticosteroid receptor types are imbalanced for a prolonged period of time (9).

The term “allostasis” has been coined to refer to the need to maintain physiological stability (homeostasis) in the face of continuously changing demands from the environment (261). Hence, allostasis describes a labile equilibrium (see *Box 1*) and is linked to unpredictability and uncontrollability as characteristic features of a severe psychological stressor. The term “allostatic load” refers to the cost of coping with the changing demands and designates the “wear and tear” that is exerted on organs and tissues under chronic stress. Cumulatively, allostatic load predisposes to an increased vulnerability to disease (268). The allostatic load model of chronic stress includes the dysregulation of the GC response, which may compromise adaptation and resilience to stressful stimuli. Chronic elevations of GC levels at the time of the normal trough of the rhythm and the resulting flattening of the circadian rhythm are hallmarks of “allostatic load.” The mediating role of misalignment of central and peripheral oscillators in increasing disease risk is only beginning to be appreciated.

Several human studies have examined whether there is a daily variation in the cortisol response to acute exposure to experimental stressors, such as physical exercise (269) and psychosocial stressors (248). The most common hypothesis motivating these studies was that the stress response would be lower in the morning, when basal activity is high, than in the evening and early part of the night, when basal activity is low. Pharmacological studies investigating the impact of time of day on the pituitary-adrenal axis response to CRH stimulation have generally found that the cortisol response is indeed higher in the afternoon or evening than in the morning, whereas the ACTH response is similar at both times of day. The findings are consistent with the existence of a rhythm of adrenal sensitivity to GC stimulation where sensitivity is higher in the evening, as demonstrated in several studies of timed exogenous ACTH administration. When low to moderate intensity physical exercise was used as a stressor, the findings regarding an impact of time of day have been mostly negative, despite the fact that physical performance is typically better in the afternoon and early evening than in the morning. However, in studies of high-intensity exercise, the impact on cortisol levels was clearly highest in the evening (269). It is thus possible that the impact of exercise on GC release is dependent on both the psychological effort and metabolic demands of the physical activity. The HPA response to a psychological stressor has been best studied using the TSST, a widely used standardized laboratory stress protocol that induces a cortisol elevation in a majority of research participants (248). The results of an analysis of 180 adults who were submitted to the TSST between 9:45 am and 7 pm did not detect significant time of day variations in the response of free salivary cortisol, total plasma cortisol, or plasma ACTH. In summary, whereas increasing numbers of individuals are involved in voluntary physical exercise in the evening or exposed to psychological stress associated with fast paced video games, it remains unclear whether these activities are consistently associated with increased GC secretion at the time of the habitual nadir, with potential adverse effects on the synchronization of central and peripheral circadian clocks.

### B. Metabolic regulation

GCs were originally named for their ability to influence glucose metabolism. To mobilize energy to respond to a stressor, acute elevations in GC promote glucose production from the liver after gluconeogenesis (270), release of free fatty acids from white adipocyte stores (271), and protein catabolism (272). Under normal conditions, the acute elevation of GC levels toward the end of the rest period frees energy from internal stores for the upcoming activity period. The main fuel for the gluconeogenetic machinery is pyruvate derived from the degradation of glucogenic amino acids. As a consequence, chronically elevated cortisol levels promote muscle degradation (273) and elevate blood glucose levels (274). Because cortisol can also positively stimulate the maturation of adipocytes from precursor cells (275, 276), it is possible that chronic cortisol elevation, eg, during repeated stress, may actively promote the deposition of energy substrates in white adipose tissues, thus predisposing to adiposity (277).

**Figure 10.**
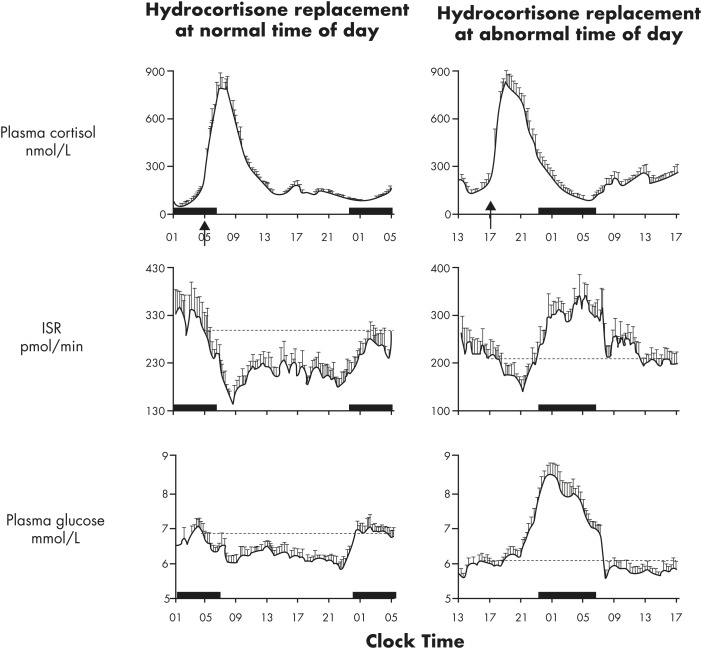
Mean (+SEM) profiles of plasma cortisol (top panels), insulin secretion rates (ISR, middle panels), and plasma glucose (lower panels) in nine healthy young men each of whom participated in four studies performed in randomized order. In all four studies, endogenous cortisol levels were suppressed by metyrapone administration, and caloric intake was exclusively in the form of a constant glucose infusion. Dark horizontal bars represent the scheduled sleep periods. The daily cortisol elevation was restored by oral administration of hydrocortisone (or placebo—data not illustrated) either at the normal time of the circadian peak (5 am, left panels) or 12 hours out of phase (5 pm, right panels). Vertical arrows show the timing of hydrocortisone administration in each study. Horizontal lines on the ISR and glucose graphs show, respectively, the mean ISR and glucose levels at the time of hydrocortisone ingestion to facilitate the visualization of post-hydrocortisone changes. The initial effect of the hydrocortisone-induced cortisol pulse was a short-term inhibition of insulin secretion without concomitant glucose changes, and the magnitude of this acute effect was similar in the evening and in the morning. At both times of day, starting 4–6 hours after hydrocortisone administration, there was a delayed hyperglycemic effect that was minimal in the morning but much more pronounced in the evening, when it was associated with a robust increase in insulin secretion. [Redrawn from L. Plat et al: Metabolic effects of short-term elevations of plasma cortisol are more pronounced in the evening than in the morning. *J Clin Endocrinol Metab.* 1999;84(9):3082–3092 (279), with permission. © The Endocrine Society.]

**Figure 11.**
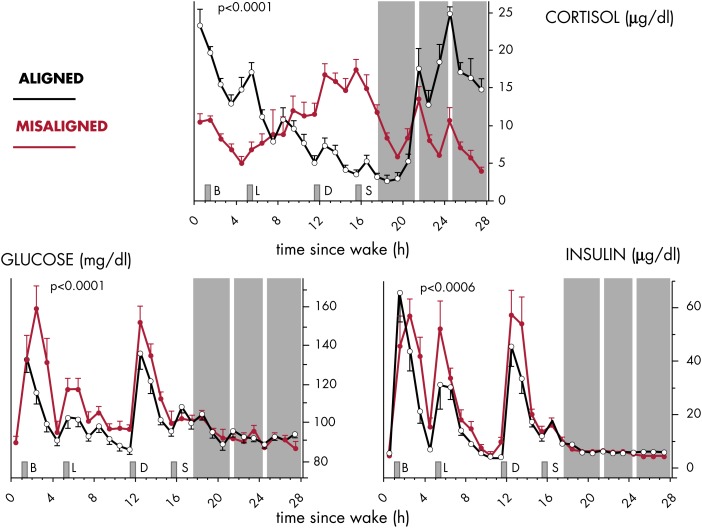
Association between the misalignment of the cortisol profile and reduced glucose tolerance despite increased insulin levels. Mean (+SEM) 28-hour profiles of plasma cortisol (top), plasma glucose (lower left) and serum insulin (lower right) in 10 healthy adults who participated in a 10-day laboratory “forced desynchrony” protocol, where sleep (shown by the large vertical gray shaded areas) and meals (shown by the small vertical gray shaded areas) were scheduled on a recurring 28-hour cycle, a periodicity out of the range of entrainment of the central circadian pacemaker. Subjects ate four meals (designated as breakfast, B; lunch, L; dinner, D; and snack, S) during each 28-hour “day.” Curves shown in black represent the profiles observed when the sleep-wake and meal schedules were aligned with the 24-hour cycle, whereas curves shown in red represent the profiles observed when the 28-hour “day” was 12 hours out of phase with the normal 24-hour day. Cortisol profiles were inverted during circadian misalignment, demonstrating no adaptation to the 28-hour schedule (as expected from a rhythm controlled by the circadian pacemaker). The postprandial glucose and insulin responses were significantly increased as compared to normal alignment, and the differences were also clinically significant. [Redrawn from F. A. Scheer et al: Adverse metabolic and cardiovascular consequences of circadian misalignment. *Proc Natl Acad Sci USA.* 2009;106(11):4453–4458 (23), with permission. © National Academy of Sciences.]

As summarized in Table 2, peripheral clocks are present in all tissues that play a major role in glucose metabolism, including the *β*-cell, the liver, skeletal muscle, and adipose tissue, and all these tissues express type II GR. Misalignment between the rhythm of circulating GC levels and peripheral clocks is thus expected to have important adverse metabolic effects, which have indeed been observed in a number of animal studies (143, 278). In humans, direct experimental evidence was obtained in a placebo-controlled study where the 24-hour rhythm of cortisol concentrations was flattened by repeated administration of metyrapone, and the peak of the rhythm was replaced by a single dose of hydrocortisone or placebo given either in the early morning, near the normal peak time, or in the early evening, near the normal trough time (279). Figure 10 illustrates the main findings. Starting 4 to 6 hours after hydrocortisone administration, glucose levels were higher than under placebo at both times of day; but this hyperglycemic effect was barely detectable when hydrocortisone was given in the morning, whereas it represented a more than 20% increase in the evening. In the evening, but not in the morning, the elevation of glucose was associated with a robust increase in plasma insulin and insulin secretion rates. The report appeared in 1999 (279), thus before the identification of peripheral circadian clocks and their relationship to metabolism, and it acknowledged that the mechanisms underlying the evening vs morning differences in metabolic effects of hydrocortisone “remain to be elucidated.” More recently, an extended laboratory study in healthy volunteers examined the metabolic effects of circadian misalignment by imposing a 28-hour sleep-wake cycle with four meals per 28 hours (23). Figure 11 compares the profiles of cortisol, glucose, and insulin observed when the subjects had their habitual 24-hour cycle with those observed when they were sleeping and eating 12 hours out of phase with the normal schedule. Circadian misalignment resulted in elevated postprandial glucose levels despite increased insulin release, suggestive of a state of insulin resistance. In laboratory studies of recurrent sleep restriction, an elevation of evening cortisol levels has been observed and proposed as one ofthe mechanisms mediating the concurrent reduction in insulin sensitivity (280, 281).

### C. Cardiovascular function

Circadian rhythmicity is present at multiple levels in the cardiovascular system. Heart rate drops by 15 to 25 bpm during the first half of nocturnal sleep, and this slowing of the heart rate is only partly related to the change in behavioral state. Absence or dampening of the nocturnal dipping of blood pressure, normally a 10–20% reduction from daytime values, is a predictor of adverse cardiovascular outcomes and mortality (282). A well-documented study that assessed the temporal profile of blood pressure under conditions that dissociated behavioral and environmental influences from inputs from the endogenous circadian system showed that there is an intrinsic circadian rhythm in blood pressure that peaks around 9 PM and contributes to the magnitude of nocturnal dipping by 3 to 6 mm Hg for systolic blood pressure and 2 to 3 mm Hg for diastolic blood pressure (283). The risk of adverse cardiovascular events, including sudden cardiac death, myocardial infarction, and syncope, has a robust daily peak in the midmorning, typically around 10 AM. Moreover, several recent studies have observed a circadian variation of infarct size in ST-segment elevation myocardial infarction patients according to the time of day of the onset of the acute myocardial infarction (284). Multiple mechanistic pathways have been identified to explain this 24-hour rhythm in the incidence of adverse cardiovascular events, including a circadian rhythm in platelet activation and in prothrombotic plasminogen activator inhibitor-1 independent of the sleep-wake cycle (285, 286), a circadian rhythm in cardiac vagal modulation and in cardiovascular reactivity to exercise and postural stress (287, 288), and an endogenous circadian-dependent rhythm in a scale-invariant pattern of heartbeat dynamics with a morning peak suggestive of increased cardiac vulnerability (52).

Importantly, most of the studies exploring the causal mechanisms of the morning peak in adverse cardiovascular events have used experimental strategies that dissociate the behavioral sleep-wake and rest-activity cycle from the central circadian signal. Overall, the findings are consistent with the findings of prospective epidemiological data showing that misalignment of behavioral rhythms relative to the endogenous clock as occurs in shift workers is associated with an increased incidence of cardiovascular disease (289).

The existence of a circadian clock in cardiac tissue is well established. Based on the response of tissue cultures from mice carrying bioluminescent reporters of the core clock genes, it appears that both the amplitude and the phase of the atrial circadian rhythm of *PER2::LUC* is affected by dexamethasone treatment as well as by the timing of medium treatment. Furthermore, the phase response was different from that observed in the liver, and *Per1* and *Per2* peaked approximately 3 hours earlier in the liver than in the heart (220), suggesting that a normal rhythm of GC signaling may be an important mechanism to entrain the circadian clock in cardiomyocytes and to maintain an optimal internal phase relationship with other peripheral clocks. Consistent with this concept, induction of the GC-sensitive *Per2* in mice during myocardial ischemia was associated with a switch from fatty acid to glucose metabolism with cardioprotective effects, including a smaller infarct size (290).

### D. Immune function

From a pharmacological perspective, one of the most interesting aspects of GCs is their high immuno-suppressive potential (291). Cortisol prevents proliferation of T cells by rendering the T cells resistant to In-terleukin-1 and unable to produce the T-cell growth factor (292). It further has a negative-feedback effect on Interleukin-1 expression and release in monocytes (293). GCs promote the redistribution of lymphocytes from the intravascular compartment to the lymph nodes, spleen, and bone marrow (294). Actions on *β*-cells include inhibition of Ig biosynthesis through the nuclear factor-*κ*B pathway and induction of apoptosis. Furthermore, cortisol inhibits monocyte differentiation and macrophage phagocytosis and cytotoxic activity (295).

It is well established that multiple components of the immune system, from both the innate and adaptive systems, undergo robust circadian oscillations (296). Collectively, these oscillations are thought to optimize the response of the host to pathogen encounters and to promote recovery. The circadian organization of the immune system impacts vulnerability to disease, disease severity, and efficacy of therapeutic interventions. The best-characterized circadian rhythm in the immune system is that of the number of circulating leukocytes in blood, which peaks during the resting phase (nocturnal sleep in humans) for most subtypes but during the active phase for some of them. Leukocytes that are most abundant in blood during the night are negatively regulated by GC levels. The early morning rise of cortisol facilitates the redistribution of these cells to the bone marrow, with a delay of approximately 3 hours.

Autonomous circadian clocks have been identified in a number of immune cells, including macrophages (297) and lymphocytes (298, 299). Recent work has identified a circadian clock in epithelial club cells of the mouse lung as well as an interaction between this clock and GCs that could explain the known rhythmic variations in symptom severity and lung function in inflammatory lung diseases such as chronic obstructive pulmonary disease and asthma (218). In normal mice exposed to infection, there is a circadian variation in neutrophil recruitment to the lung that is driven by the rhythmic expression of the chemokine CXCL5, itself under GC control. Genetic ablation of the circadian clock within bronchoalveolar cells results in enhanced and arrhythmic CXCL5 expression, despite normal rhythmicity of circulating GC and of GR occupancy. These genetically manipulated animals no longer respond to the anti-inflammatory action of dexamethasone.

### E. Arousal

The regulation of physiological and psychological arousal across the 24-hour cycle is one of the most common interpretations of the functional significance of the rhythm of HPA activity. The rapid rise of HPA activity and GC levels after the first few hours of sleep is generally thought to prepare the brain for cognitive activity and to prepare the periphery, including the liver, muscle, and adipose tissue, for impending meal intake and physical activity (107). As the day progresses, the slow decline toward evening quiescence prepares instead for the initiation of sleep. An important interaction in the control of arousal is the reciprocal excitatory feedback between HPA activity controlled by the PVN and norepinephrine release from the brainstem locus coeruleus. CRH activates the locus coeruleus to release norepinephrine, which in turns activates hypothalamic CRH.

Appropriately timed normal sleep enhances the amplitude of the rhythm of HPA activity and stabilizes its phase (illustrated in Figure 8). Under normal conditions, the initiation of sleep occurs when HPA axis activity is near nadir levels and the circadian rise of ACTH and cortisol occurs roughly 2–3 hours after sleep onset, in parallel with an increased propensity for rapid eye movement (REM) sleep. When sleep is initiated at an abnormal time of day, an acute inhibitory effect is exerted on GC levels, and this appears to be mediated by deep non-REM (NREM) sleep. Nighttime awakenings coincide with cortisol pulses and CRH release and are followed by a temporary inhibition of cortisol secretion (300). Upon final morning awakening, cortisol levels generally show a rapid rise that may continue for about 1 hour. This phenomenon is called the cortisol awakening response and appears to be independent of circadian regulation (246). The combination of the sleep onset inhibition of HPA activity with the post-awakening stimulation enhances the amplitude of the human cortisol rhythm by about 15% (113).

### F. Memory and cognition

The importance of circadian fluctuations in GC levels for memory and learning has been highlighted by a recent study by Liston et al (301). The authors studied transgenic mice with fluorescent neurons in the motor cortex of the brain, enabling them to study the effects of circadian GC exposure on neuronal structure in vivo. High levels of GCs at the time of learning (for example, during the evening circadian peak in this nocturnal species) were associated with improved learning of motor skills and increased spine formation in cortical neurons. This process was mediated via rapid nongenomic GR action. Low levels of GCs during circadian troughs, however, were required for memory consolidation in the days after learning. This stabilization process depended on the “pruning” of older spines by MR-mediated changes in gene expression (301). The study by Liston et al (301) used the synthetic GC dexamethasone to suppress HPA axis activity and circulating corticosterone levels. Dexamethasone targets the pituitary corticotrophs (302). The brain is resistant to penetration by dexa-methasone because it is a substrate for multidrug resistance P-glycoprotein at the level of the blood-brain barrier (303). The brain and in particular the MR is therefore depleted of corticosterone. Intermittent administration of corticosterone to these dexamethasone-treated animals appeared to be a prerequisite to promote learning-dependent plasticity (304).

In line with the work by Liston et al (301), Rimmele et al (305) have shown that blocking the circadian rise of cortisol in the early morning in humans using high-dose metyrapone impaired memory retrieval of emotional and neutral texts and pictures learned 3 days earlier. Of note, the suppression of cortisol levels by metyrapone is achieved by inhibition of the enzyme 11-*β* hydroxylase, resulting in high levels of 11-deoxycortisol, a steroid that has mineralocorticoid activity. A placebo-controlled study showed that cortisol infusion during a daytime nap as compared to a period of wakefulness at the same time of day had opposite effects on consolidation of memory of temporal sequence (306). Another study suggested that memory formation during sleep may be impaired by both insufficient MR activation and GR overactivation (307). Overall, the physiological rhythm in circulating GCs appears to improve memory function. Fluctuations in GR and MR signaling regulate plasticity in memory-encoding brain regions. This might have important implications for the timing for GC therapy. These findings also provide a possible explanation for the impairment of cognitive function observed in patients with Cushing’s syndrome (see *Section VI. D*) and in individuals with nonchronophysiological synthetic GC replacement therapy (308).

## VI. Conditions of Abnormal Circadian Rhythmicity of Circulating Glucocorticoids

Three types of mechanisms resulting in abnormal GC rhythms can be distinguished. First, the master circadian clock in the SCN is altered. The best-documented condition falling in this category is aging. Second, the master circadian clock is not altered, but the GC rhythm is disrupted by behavioral or pathophysiological factors. This category includes shift work, an increasingly common condition, sleep disturbances, clinical sleep disorders, fatigue, and chronic stress conditions. Third, the master clock is not altered, but the GC rhythm is profoundly abnormal due to Cushing’s syndrome, adrenal insufficiency, or chronic treatment with synthetic GC.

### A. Aging

With advancing age, a number of behavioral and physiological human circadian rhythms are disrupted. There is good evidence that the reduced amplitude of multiple daily cycles and the increased prevalence of sleep disturbances in older adults are associated with neuronal degeneration in the SCN, the master clock (309). However, the intrinsic period of the human circadian pacemaker does not seem to be affected by age (310) . The senescence of the circadian system is more pronounced in Alzheimer’s disease than in normal aging (311, 312).

**Figure 12.**
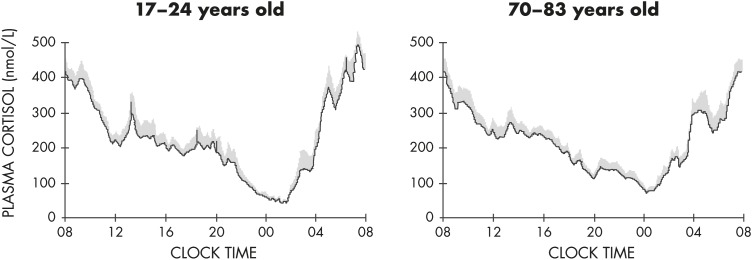
Impact of age on the 24-hour profile of plasma cortisol in healthy nonobese men. Data are shown at each time point as mean + SEM. The two age groups included eight men that were matched for body mass index. Note the higher nadir level, the reduced amplitude of the overall rhythm, and the earlier onset of the early morning rise in older participants. The overall disruption of the 24-hour cortisol profile is likely to contribute to and/or exacerbate age-related metabolic, immune, and cognitive deficits. [Redrawn from E. Van Cauter et al: Age-related changes in slow wave sleep and REM sleep and relationship with growth hormone and cortisol levels in healthy men. *JAMA.* 2000;284(7):861–868 (313), with permission. © American Medical Association.]

Commonly observed changes of HPA axis activity in older adults indeed include reduced amplitude of the 24-hour cortisol profile, mostly due to an elevation of the nadir, and decreased reactivity and resilience to stressors. Figure 12 illustrates the mean 24-hour profile of plasma cortisol from a group of 17- to 24-year-old men as compared to that collected in a group of healthy nonobese 70- to 83-year-old men. The dampening of the rhythm, the higher nadir level, and earlier timing of the onset of the circadian rise are readily apparent. Thus, in old age, the 24-hour rhythm of GC provides a weaker synchronizing signal for central and peripheral clocks. With aging, evening cortisol levels increase progressively in both men and women (313), and this may lead to an overall increase in mean 24-hour levels (314). Interestingly, the chronology of appearance of this disturbance of the cortisol rhythm is similar to that observed for the age-related decrease in REM sleep, with both alterations developing from midlife to old age (313). The age-related elevation of the cortisol nadir is consistent with a reduced resilience of the HPA axis to a challenge, with slower recovery, and could facilitate the development of central and peripheral disturbances associated with GC excess, including memory deficits, insulin resistance, and osteoporosis. A recent metaanalysis of HPA function and physical performance shows that a greater decline of HPA activity across the daytime (ie, likely to reflect a greater amplitude of the rhythm) is associated with better physical performance in later life (315). These findings are consistent with observations from the Whitehall II study where “raised curves of saliva cortisol” with a flatter pattern of release were associated with older age and impaired physical functioning (316).

### B. Shift work

Circadian disturbances triggered by conflicting synchronizing cues (eg, rotating shift work, recurrent jet lag) are now considered as pathogenic when recurring on a chronic basis. Irrespective of their work schedule, the circadian system of shift workers rarely adapts to the behavioral rest-activity cycle because the vast majority of shift workers revert to daytime activities during non-work days. Shift work is associated with a higher incidence of obesity, cardiovascular disease, sleep disturbances, impaired immune function, and cancer (317, 318). Of note, most of these conditions may also arise from disruption of the circadian system—at least in animal models (319, 320). Shift work disorders usually manifest only after several years of shift work, but this may be partly due to the fact that individuals who are shift work intolerant and prone to quickly develop adverse effects tend to return to regular daytime working schedules. Under conditions of shift work, central and peripheral oscillators dissociate, which leads to a misalignment of daily rhythms in physiological functions (321–324), including endocrine axis regulation and melatonin. Synchronization during shift work can be improved by melatonin supplementation, adequately timed exposure to light and/or dark, and manipulation of the timing and composition of food intake (321, 325–329). Depending on the shift schedule, GC rhythms can partly adapt. Urinary cortisol in night shift workers has been reported to rise by 16% during daytime sleep and to decrease by 13% during nighttime sleep on the off-nights. Morning serum cortisol is reduced compared to day shift workers (330). It remains to be shown whether this is primarily an adaptation to shifted activity and/or meal timing or reflects increased stress exposure during work hours. Given the role of GCs in systemic circadian alignment (187, 190), manipulating the 24-hour cortisol profile in shift workers may have preventive potential against some of the adverse health effects of shift work (227). In a mouse model, this strategy was shown to accelerate adaptation to jet lag (190).

### C. Sleep disturbances, clinical sleep disorders, and fatigue

There is a bidirectional relationship between HPA activity and sleep. On the one hand, excessive HPA activity promotes sleep fragmentation, resulting in shallow and insufficient sleep. On the other hand, insufficient sleep and poor sleep quality can result in elevated GC levels, particularly in the late afternoon and early evening when the HPA axis is normally quiescent to facilitate sleep onset and maintenance. Under chronic conditions, this vicious cycle can be difficult to interrupt. In laboratory studies, sleep deprivation, whether total or partial, acute or recurrent, has been associated with an elevation of HPA activity in the late afternoon and evening hours (117, 280, 281, 331). Thus, sleep loss appears to delay the return to quiescence of the HPA axis, similar to aging (332). Chronic insomnia with an objective reduction of total sleep time is associated with higher cortisol levels across the later part of the day and the night (165). This relative ambient evening hyper-cortisolism appears to play a role in the effects of sleep deprivation on gene expression in the brain (333). An elevation of daytime cortisol secretion has been observed in some (334, 335), but not all (336), studies of patients with obstructive sleep apnea. Well-documented studies have found positive effects of continuous positive airway pressure treatment (185, 334, 335, 337). It has been proposed that dysfunctional HPA axis activity (eg, by chronic stress or due to adrenal hyperfunction; see *Section VI. D and E*) may play a role in promoting sleep disorders such as insomnia, but that HPA axis dysfunction may also be the consequence of a sleep disorder, as could be the case for obstructive sleep apnea (300, 338, 339). Of note, multiple reports have documented alterations of the cortisol rhythm in obesity and type 2 diabetes (340, 341). However, none of these studies controlled for the presence of obstructive sleep apnea. A study using frequent sampling of saliva melatonin under dim light conditions found no difference in the timing of nocturnal melatonin release in patients with sleep apnea as compared to controls (342), suggesting that the phase of the master clock is not affected by this sleep disorder.

Because of its lipophilic nature, cortisol readily penetrates the blood brain barrier and exerts numerous effects, in particular on sleep regulation. Conditions of hypercortisolism, whether due to a pathology or due to exogenous administration, are therefore associated with sleep disturbances. In Cushing’s syndrome, deep NREM sleep is reduced, but interestingly, in patients with pituitary-dependent disease, adrenal secretory activity is still initiated predominantly during NREM sleep, suggesting the persistence of a link between pulsatile cortisol release and the ultradian NREM-REM cycle (339, 343). Exogenous GCs, both natural and synthetic, taken near bedtime inhibit REM sleep. Hydrocortisone appears to stimulate slowwave sleep, whereas synthetic GCs such as dexamethasone inhibit slow-wave sleep (339, 344, 345).

Sleepiness is a condition where there is increased pressure to sleep and sleep will be easily initiated when an opportunity presents. In contrast, fatigue describes a condition of tiredness, exhaustion, and/or low energy but without increased pressure to sleep (346). Chronic fatigue syndrome is often associated with alterations in HPA axis activity such as mild hypocortisolism, attenuated amplitude of cortisol rhythm, enhanced negative feedback to the HPA axis, and blunted HPA axis responsiveness (347). A similar constellation of alterations of GC regulation may be seen in PTSD (249). In fibromyalgia, one study observed a slower decline of cortisol levels across the daytime period, with elevated concentrations (348), but another report found no evidence for disturbances of the 24-hour GC profile (349). All these conditions frequently include sleep disturbances, but there is no evidence for alterations in central pacemaker function. Whether eliminating or reducing the sleep disturbances could reduce the severity of the other neuropsychiatric and somatic symptoms is still an open question.

### D. Cushing's syndrome, adrenal insufficiency, and glucocorticoid therapy

#### 1. Cushing's syndrome

**Figure 13.**
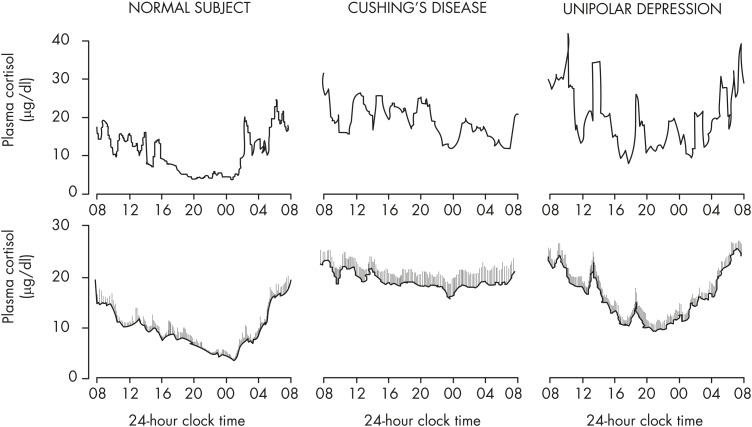
Comparison of the 24-hour profile of plasma cortisol in normal nonobese adults (left), patients with pituitary-dependent Cushing’s disease (center), and patients with untreated major depression of the unipolar subtype (right). For each condition, a representative example is shown in the top panel and mean (+ SEM) profiles from eight to 10 subjects are shown in the bottom panel. [Redrawn from E. Van Cauter: Physiology and pathology of circadian rhythms. In: Edwards CW, Lincoln DW, eds. *Recent Advances in Endocrinology and Metabolism.* Vol. 3. Churchill Livingstone, Edinburgh, UK, 1989:109–134 (435), with permission.]

Adrenal hyperactivity, caused by tumors of the pituitary or the adrenal cortex, is characterized by central obesity, hirsutism, moon face, plethora, several cardiometabolic disturbances (including hypertension, glucose intolerance, and dyslipidemia that result in an increased cardiovascular risk), osteoporosis, and multiple psychiatric abnormalities. Adrenal hyperactivity is referred to as Cushing’s *syndrome,* irrespective of the etiology (350). Cushing’s *disease* refers to secondary hypercortisolism derived from ACTH overproduction in the pituitary (351). A low amplitude circadian variation may persist in some patients with pituitary-dependent Cushing's disease, but overall circadian modulation is absent (Figure 13, middle panel). Cortisol pulsatility is blunted in about 70% of patients with Cushing’s disease, suggesting autonomous tonic secretion of ACTH by a pituitary tumor. However, in about 30% of these patients, the magnitude of the pulses is instead enhanced (352). These “hyperpulsatile” patterns could be caused by enhanced hypothalamic release of CRH or persistent pituitary responsiveness to CRH.

In patients with primary adrenal Cushing’s syndrome, increased cortisol secretion appears to result from both increased basal secretion and increased pulse frequency (353). A partial persistence of cortisol rhythmicity has rarely been observed, but when present, it could be mediated by the neural pathway between the SCN and the adrenal cortex (106). SCN function is not affected by the hypercortisolism of Cushing’s syndrome, consistent with the absence of GR expression in this neural tissue (Table 1). Circadian phase, as assessed by dim light melatonin onset (DLMO), has been examined in patients with Cushing’s syndrome of both adrenal and pituitary origin, and no differences from controls were detected (354). It has been suggested that alterations in the 24-hour GC rhythm may actually serve as an early preclinical marker for adrenocortical tumors (119, 355, 356). This is also the case for pituitary ACTH-secreting tumors, where one of the first clinical abnormalities is the elevation of the nocturnal nadir of cortisol.

The absence or the marked dampening of cortisol circadian variations in Cushing’s syndrome has obvious diagnostic implications because the time of day when blood samples are obtained has to be taken into account in the evaluation of the result. Differentiation between normal and pathological levels is greatly improved by adequately selecting the sampling time because the overlap between normal individual values and values in patients with Cushing’s syndrome is minimal during a 4-hour interval centered around midnight (113). The reduced amplitude of the circadian cortisol rhythm associated with higher nadir levels is, in clinical practice, an early marker of hypercortisolism in humans, and the evaluation of cortisol levels during the normal nadir of the rhythm is a common screening test to diagnose Cushing’s syndrome (357). Moreover, the gradual recovery of a normal circadian variation in circulating cortisol levels is also a clinical marker of the cure of Cushing’s syndrome, reflecting a normalization of the entire neural regulation of the HPA axis. In contrast, the persistence of a dampening of the cortisol rhythm, even when absolute cortisol concentrations in blood or urine are within the normal range, usually predicts a relapse of the disease (358).

Lastly, Cushing’s syndrome may also result from ectopic ACTH secretion from a nonpituitary tumor. In this case, ACTH release from the tumor is not under circadian control, and reproducible 24-hour variations in GC are not detectable.

#### 2. Adrenal insufficiency

Primary hypocortisolism, or Addison’s disease is mainly due to autoimmune defects (70% of all cases), but it can also be caused by infections such as tuberculosis, HIV, fungi, or cytomegalovirus that can result in direct destruction of the adrenal cortex (359). In many cases, hypocortisolism is only diagnosed when patients present with an acute crisis during an intercurrent disease. Daily rhythmicity of circulating ACTH and cortisol is generally absent or blunted in Addison’s patients (360). Severe fatigue is present in about half of the patients and is associated with sleep disturbances (339, 361). Basal concentrations of GCs are a poor diagnostic indicator of Addison’s disease. Thus, direct ACTH stimulation tests are necessary to assess adrenal functionality. Secondary partial hypoadrenalism, due to ACTH deficiency, is more difficult to detect, and its diagnosis often requires indirect measures such as the insulin tolerance test (359). In patients with either primary or secondary adrenal failure, inadequate replacement of GCs may lead to hypotension, diarrhea, abdominal pain, weight loss, poor stress responses, and electrolyte abnormalities. Conversely, overdosing with GCs without respecting the physiological daily rhythm will promote Cushing’s-like features such as glucose intolerance, hypertension, and cardiovascular disease, as well as osteoporosis and disturbances of the immune system (362–365). There is evidence that the commonly used hormonal replacement therapy in patients with adrenal failure does not mimic the physiological circadian rhythm of cortisol secretion, resulting in a nonphysiological plasma cortisol profile, as well as a risk of over- or under-replacement, associated with increased morbidity and premature mortality (366). Thus, as will be discussed further in *Section VII,* mimicking the circadian profile of cortisol blood levels may help to minimize side effects and increase the quality of life in patients with adrenal insufficiency (367, 368).

#### 3. Glucocorticoid therapy

About 1% of the Western world receives therapy with potent synthetic GCs resulting in supraphysiological exposure. During treatment, patients suffer a 2-fold higher risk of depression; a 4-fold higher risk of mania, delirium, confusion, or disorientation; and nearly a 7-fold higher risk of suicide (369). Moreover, cessation of GC therapy may result in enduring psychiatric complaints (370). One possible cause of GC-induced psychopathology is the suppression of the episodic secretion of the endogenous GC over the 24-hour cycle and after stress. Continuous exposure to synthetic GCs may cause desensitization and reduced responsiveness of GR-dependent neuronal networks. When synthetic GCs such as dexamethasone, prednisone, and related steroids that have limited brain penetration are used, the depletion of endogenous hormone levels may cause a hypocortisolemic state in the brain, in particular with respect to the lack of MR and episodic GR activation (304, 371). Hence, a severe MR:GR imbalance may develop during synthetic GC therapy. Replacement with cortisol mimicking the circadian pattern to restore the receptor balance may therefore help to ameliorate the side effects of potent synthetic GCs (36).

### E. Depression and post-traumatic stress disorder

Two types of depression are distinguished in the Diagnostic and Statistical Manual of Mental Disorders, 5th edition (DSM-5), namely melancholic depression and atypical depression. The two subtypes are characterized by opposite alterations in HPA axis activity and in circadian expression of the severity of the symptoms (372). Hypercortisolism with persistent circadian rhythmicity and increased pulsatility is found in a majority of severely depressed patients, particularly those with melancholic features who have more severe symptoms in the morning (373–375). Cortisol profiles are illustrated in Figure 13 (right panels). These patients do not develop the clinical signs of Cushing’s syndrome despite the high circulating cortisol levels, possibly due to the GC resistance characteristic for these patients. The quiescent period of cortisol secretion is shorter and more fragmented, and it often starts later and ends earlier than in normal subjects of comparable age. These alterations could reflect the impact of sleep disturbances (low amounts of deep slow-wave sleep and reduced latency to REM sleep) as well as an advance of central circadian phase. When a clinical remission of the depressed state is obtained, the hypercortisolism and the alterations in the quiescent period disappear, indicating that these disturbances are state-dependent, rather than trait-dependent (376).

Atypical depression is in some of its symptomatology the opposite of melancholic depression. The symptoms display a 24-hour variation, with the worst presentation in the evening. Atypical depression is characterized by lack of energy, fatigue, hypersomnia, and unexplained weight gain. The activity of the HPA axis is reduced rather than enhanced, reflecting a hypofunction of CRH neurons in the PVN resulting in reduced circulating levels of cortisol (372). Blunted HPA activity can be associated with a dampening of the circadian variation (375, 377). It is often difficult to distinguish cause and effect in cases of atypical depression. Chronic stress or traumatic events may have led to elevated cortisol levels and blunted circadian rhythmicity, which then triggered the disease outbreak. On the other hand, the social disapproval that is often perceived by sufferers of mental disorders added to the sleep-wake problems may become stressful in a depressive person, thus interfering with normal circadian cortisol responses. Given the role of GCs in regulating sleep (see *Section VI.* *C*), it may be of clinical interest to determine the extent to which stabilization of the circadian cortisol profile and correction of sleep disturbances may ameliorate the symptoms (378, 379).

Circadian secretion of cortisol in chronically stressed humans is also dampened due to higher levels during the habitual daily nocturnal trough, an alteration identified as a risk factor for insulin resistance, obesity, and memory impairment (380). A meta-analysis of 47 studies analyzing cortisol effects in PTSD found lower basal morning cortisol levels in individuals with PTSD than in controls not exposed to trauma, irrespective of the presence of major depression as a comorbid factor (381). Afternoon and evening levels were also lower in PTSD subjects with comorbid major depression who had higher levels of cortisol at that time of day. Although findings are highly heterogeneous, it appears that some dampening of the circadian variation of cortisol may be present in many cases, and this is most clearly seen in those with a more severe condition (382). The paradoxically lower cortisol levels found in multiple studies of PTSD are interpreted as reflecting an increased sensitivity to negative feedback regulation of HPA activity (249). Lower cortisol levels were also found in PTSD females, eg, after physical or sexual abuse. These findings suggest that enhanced HPA negative feedback regulation may be a marker of trauma exposure rather than a specific mechanism of vulnerability for PTSD, whereas lower daily cortisol output may be associated with PTSD in particular (381, 382). Of note, the timing of the onset of nocturnal melatonin release, a marker of central circadian phase, is similar in subjects affected by PTSD and in well-matched controls (383).

### F. Eating disorders

Abnormal eating behavior manifests in pathologies such as anorexia nervosa, bulimia nervosa, night- and binge-eating syndromes, all of which display signs of circadian disruption. Anorexia nervosa is defined by a general restriction of food intake resulting in a low body weight, whereas a hallmark of bulimia nervosa is excessive bouts of uncontrolled eating followed by purging. Both patient groups often show confounding symptoms of depression, which has been closely associated with disruption of cortisol rhythms (see *Section VI.* E). In anorectic patients, total plasma cortisol levels as well as free saliva cortisol levels are elevated; the circadian variation persists but can be dampened (384, 385). Interestingly, cortisol perturbations can be reversed after refeeding, perhaps suggesting that the hypercortisolism is due to metabolic stress as a consequence of low adiposity (384, 386, 387).

Binge-eating disorder is very close to bulimia nervosa but does not involve postprandial purging. Few studies have characterized endocrine rhythms in these patients, and the results are somewhat conflicting. Mechanisms underlying the night-eating syndrome are also unclear. One possible pathway is that the night-eating behavior itself causes misalignment in the circadian clock system, culminating in metabolic perturbations. Vice versa, circadian disruption may promote the development of night-eating syndrome, eg, as a consequence of altered cortisol and melatonin rhythms. In patients suffering from night-eating syndrome, the nocturnal melatonin peak is delayed (388), resulting in elevated morning melatonin levels (389). Peak cortisol levels are either reduced in patients with night-eating syndrome (388, 389) or increased (390).

## VII. Manipulating the 24-Hour Rhythm of Circulating Glucocorticoids to Optimize Circadian Alignment

### A. Chronopharmacology of conditions of excessive cortisol production: An untapped opportunity?

The most common disturbance of the human cortisol rhythm, observed in a wide variety of conditions, is abnormally elevated afternoon and evening levels. In the mean profiles shown in Figures 12 and 13, it can be seen that morning levels in older adults, patients with Cushing’s disease, patients with untreated major depression, and primary insomniacs with short sleep duration overlap with levels observed in normal young adults. In contrast, the evening levels are clearly elevated and, as discussed above, are likely to impair sleep duration and quality and perpetuate a feed-forward loop further compromising the return to quiescence of the HPA axis on the following day. This suggests that normalization of the 24-hour cortisol profile should target the evening period to recreate the physiological quiescent period. Acute pharmacological suppression of cortisol levels at specific times of day has been used in healthy human adults to elucidate metabolic (279) and cognitive implications of cortisol rhythmicity (305, 306, 391). These studies used metyrapone, an inhibitor of cortisol biosynthesis with a rapid onset of action and biological half-life of about 2 hours, or the GC receptor antagonist mifepristone. As expected, the dose of metyrapone needed to suppress physiological cortisol levels was much lower in the evening (500 mg) than in the morning (3 g). Pharmacological suppression of cortisol levels or action has also been explored in a variety of pathological conditions including patients with Cushing’s disease in whom hypercortisolism could not be controlled by a surgical intervention (392, 393), and more recently in patients with treatment-refractory depression (394) or type 2 diabetes (395). A recently published multicenter retrospective study assessed the effectiveness of metyrapone in controlling cortisol excess in a large series of patients with Cushing’s syndrome and concluded that metyrapone is an effective therapy for short- and long-term control of hypercortisolemia (396). Surprisingly, it seems that none of these recent trials targeted evening, rather than daytime, cortisol levels or action.

### B. Chronopharmacotherapy of adrenal insufficiency

GC replacement therapy has drastically improved survival and reduced morbidity in patients with adrenal insufficiency who, until about 1949, ie, before the era of hydrocortisone substitution, would have faced an average survival span after diagnosis of just 1–2 years (397). But still, excess morbidity and mortality of patients with adrenal insufficiency on GC replacement persists even under state-of-the-art replacement regimens. Mortality risk remains elevated more than 2-fold (33), and ample evidence documents increased long-term morbidity in patients with adrenal insufficiency of any cause, with an excess risk of cardiovascular disease (33, 398, 399), psychological disorders (400), and a significant negative impact on health-related quality of life (401, 402).

Major treatment goals of GC substitution for adrenal insufficiency include the recreation of physiological 24-hour cortisol exposure profiles in the circulation and at target organs to guarantee adequate stress reactivity, avoiding life-threatening acute adrenal insufficiency, and preventing symptoms and adverse consequences of chronic adrenal insufficiency, eg, fatigue, nausea, and weight loss (403). At the same time, care must be taken to administer as little hydrocortisone as possible to avert detrimental psychological, metabolic, and cardiovascular side effects of iatrogenic GC excess (404–406). This delicate balance between overtreatment and undertreatment of adrenal insufficiency makes reliable monitoring instruments very desirable. Current evaluation of treatment adequacy is based primarily on clinical criteria including changes in weight, blood pressure, electrolyte levels, and quality of life assessments (401, 402). A significant problem in using clinical criteria as the sole monitoring instrument and guide to treatment lies in the fact that clinical evidence for GC overdose (eg, glucose intolerance, weight gain, trophic skin changes) or GC under-replacement (eg, daytime fatigue, diarrhea, and nausea) lacks specificity. Therefore, biochemical monitoring in the form of blood (407) or salivary cortisol (408) profiles has been employed to assess the adequacy of GC replacement. The validity of using daytime profiles of salivary cortisol for monitoring GC substitution regimens is still controversial. Ceccato et al (408) found that two salivary cortisol measurements in patients with secondary adrenal insufficiency were as good as six measurements to predict the area under the curve during oral treatment with cortisone acetate. The same authors have suggested that a single measurement of saliva cortisol upon awakening can facilitate the diagnosis of adrenal insufficiency (408). In contrast, Thomson et al (409) found a significant interindividual variation in plasma and salivary cortisol profiles subsequent to oral hydrocortisone treatment for adrenal insufficiency of primary or secondary origin. Furthermore, resulting plasma and salivary cortisol levels correlated only poorly, leading the authors to conclude that salivary cortisol measurement is not a sufficient tool for monitoring the adequacy of hydrocortisone substitution. In contrast, more recent work found an excellent correlation between levels of total cortisol in plasma and free cortisol in saliva, both sampled at 20-minute intervals from early afternoon until bedtime in healthy young adults (illustrated in Figure 6) (117). In a direct comparison, Arlt et al (363), correlating a self-developed clinical score to timed cortisol serum measurements in the assessment of GC replacement quality, found that three timed cortisol serum measurements were of very limited practical value in guiding replacement therapy because cortisol levels of patients determined as under- and oversubstituted with cortisone acetate largely overlapped with those of well-replaced patients. Unfortunately, the most precise method of gauging the adequacy ofrecreating physiological, circadian systemic cortisol profiles remains highly repetitive blood or saliva sampling, which, due to cost and labor, does not represent a feasible, clinical biomarker. In sum, the existing monitoring tools are far from perfect in accurately assessing treatment quality, emphasizing the importance of characterizing new biomarkers of treatment quality.

**Figure 14.**
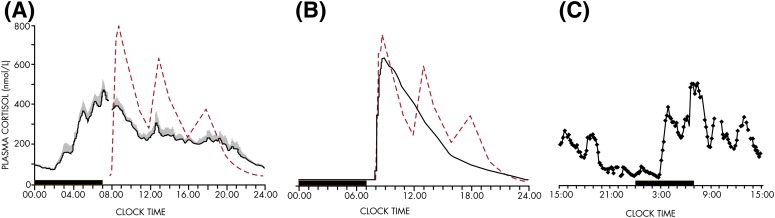
A, Comparison between 24-hour plasma cortisol profile typically achieved with optimal hydrocortisone replacement (10 mg upon awakening, 5 mg at lunch, and 5 mg at 7 pm [dinner]) in patients with adrenal insufficiency (dotted red lines) and normal cortisol levels from healthy young adults (shaded area) (data source, Refs. 313 and 416). B, Profile of circulating cortisol levels achieved by a single early morning administration of a modified-release oral hydrocortisone preparation (solid line) as compared to the profile resulting from immediate release hydrocortisone administered three times daily (dashed line). [Redrawn from G. Johannsson, et al: Improved cortisol exposure-time profile and outcome in patients with adrenal insufficiency: a prospective randomized trial of a novel hydrocortisone dual-release formulation. *J Clin Endocrinol Metab.* 2012;97(2):473—481 (416), with permission. © The Endocrine Society.] C, Example of a plasma cortisol profile obtained in a healthy subject in whom endogenous cortisol levels were suppressed by 5 days of metyrapone administration and replaced by sc hydrocortisone replacement via an infusion pump programmed to mimic circadian and pulsatile variations. [Adapted from G. M. Russell, et al: Subcutaneous pulsatile glucocorticoid replacement therapy. *Clin Endocrinol (Oxf).* 2014;81(2):289–293 (422), with permission. © Blackwell Scientific Publications.]

Conventional pharmacotherapies of adrenal insufficiency for prolonged periods of time often have adverse side effects, such as osteoporosis, altered glucose and fat metabolism, and increased cardiovascular risk, mainly due to overtreatment. Moreover, the conventional hormone replacement therapy does not significantly protect the patient against the threat of acute adrenal crisis caused by undertreatment. Therefore, optimization of GC replacement therapy remains a challenging task for endocrinologists. One promising aim to limit side effects and improve quality of life of hypocortisolic patients might be the restoration not only of physiological levels of circulating cortisol, but also of its circadian (ie, anticipating dawn as in normal subjects) and pulsatile dynamics (410–412). Current typical hydrocortisone substitution regimens consist of 15–25 mg administered orally every 24 hours, generally in two or three divided doses, and this substitution regimen is often in excess of the physiological cortisol production of healthy subjects (approximately 3 to 12 mg/m^2^ per 24 hours) (2, 3). Disadvantages of these standard replacement regimens include nonphysiological cortisol serum profiles with acute large elevations after the midday and afternoon doses as well as subphysiological cortisol concentrations before the administration of the subsequent dose (illustrated in Figure 14A). Furthermore, the vast majority of patients report that multiple daily dosing is problematic, with difficulties related to forgetting doses, particularly at midday and in the afternoon (413). Furthermore, missing a dose results in fatigue and exhaustion and taking a dose too late in the day causes sleep problems. Overall, the nonphysiological 24-hour profiles of circulating GC resulting from prescribed multiple dosing are likely to contribute to excess mortality in GC-treated patients with adrenal insufficiency (411, 414).

Thus, the improved approximation of the circadian cortisol rhythm has recently emerged as a novel and important target in the improvement of GC therapy.

Toward this goal, a dual-release oral hydrocortisone preparation has been tested and is now marketed in several countries for the treatment of adrenal insufficiency (415–417). This preparation combines a fast, immediate-release surface hydrocortisone component with a delayed-release hydrocortisone center (417). Once daily administration of this preparation shortly after morning awakening vs thrice daily standard hydrocortisone, in addition to a very likely long-term improvement of medication adherence, enabled a closer approximation of the normal daytime cortisol secretory profile, as shown in Figure 14B (415, 416). However, this dual-release hydrocortisone does not replace the rapid rise of cortisol concentrations that is normally initiated after a few hours of sleep. Nonetheless, in a study involving 64 patients with primary adrenal insufficiency, this novel therapy translated into reduced body weight, reduction in blood pressure, improved glucose homeostasis, and improved quality of life (416).

Analogous to continuous sc insulin infusion in type 1 diabetes mellitus, several studies have attempted to recreate a physiological circadian cortisol serum profile by means of continuous pump-driven sc cortisol infusion (418–421). Løvås et al (420) used continuous sc hydrocortisone infusion by programmable infusion pumps in seven patients with primary adrenal insufficiency to successfully mimic the physiological circadian cortisol secretory rhythm, thus allowing for a considerable decrease in hydrocortisone substitution requirements without negatively impacting subjective health status. In another study, continuous 24-hour sc hydrocortisone infusion was effective in normalizing ACTH plasma concentrations and stabilizing circulating glucose concentrations during the night as compared with oral hydrocortisone replacement without impinging on endogenous insulin sensitivity as measured by the glucose clamp technique (418). Refining this paradigm, another study demonstrated that pulsatile, as compared with continuous, sc hydrocortisone administration resulted in a closer approximation of physiological ultradian and circadian cortisol secretory profiles and activated a specific pattern of gene transcription and downstream neuroendocrine responses (422). An example is shown in Figure 14C. Although pump-driven continuous or pulsatile cortisol replacement therapy will be unlikely to replace standard therapy for most patients with adrenal insufficiency due to its high cost and the need for extensive patient training in handling the infusion pump, it might ameliorate morning symptoms in those patients who suffer most from severe morning fatigue. This subgroup of patients is deemed to be more responsive to the absence of the cortisol awakening response, ie, the physiological cortisol peak shortly before awakening (423). This rise in circulating cortisol levels cannot be mimicked by the currently available oral hydrocortisone formulations. A delayed-release cortisol formulation with sustained release kinetics is currently under development (for concise review, see Ref. 424). In healthy subjects, administration of this preparation late at night (15–20 mg) and in the morning (10 mg) resulted in a close recreation of the circadian profile with very low cortisol levels during the night, a physiological approximation of the early morning cortisol rise, resulting in a cortisol peak around the time of waking (425) and physiological cortisol profiles during the remainder of the day (426). A very recent phase 2 study in 16 patients with congenital adrenal hyperplasia has had encouraging results, including suppression of androgen production (427). However, both the current and proposed hormonal replacement treatments using cortisol do not result in pulsatile GC levels, which seem to be necessary, as mentioned above, for normal transcriptional and behavioral responses in the target tissues.

### C. Assessing central circadian phase and circadian alignment in humans

The onset of nocturnal melatonin secretion under dim light conditions (DLMO) is considered the most reliable marker of the phase of the master clock. Melatonin rhythms can be quantified from saliva or from plasma samples. The total overnight melatonin output can be estimated by measuring its urinary metabolite, 6-sulfa-toxymelatonin. A practical problem is the number of samples needed to get a reliable estimate of the phase of the rhythm (428, 429). Protocols to estimate the DLMO under field conditions have been developed and validated (430). The timing of the nadir of plasma cortisol levels or of the nadir of core body temperature under conditions of total sleep deprivation (eliminating the masking effect of sleep onset) are other validated markers of central circadian phase, but their acquisition is clearly more demanding than that of the DLMO. Human fibroblasts transfected with a lentiviral circadian reporter show in vitro rhythmicity, but the period is not strongly correlated with the period of circadian behavior of the donors (431, 432). Thus, this measure does not provide a reliable estimate of the circadian phase of the donor. Repeated blood samples in healthy subjects have been used to detect rhythmic expression of clock genes in blood mononuclear cells that paralleled hormonal and behavioral phase markers (223, 224). The time resolution is limited by the prohibitive cost. Another novel strategy is based on measuring rhythms of clock gene expression using repeated sampling of hair follicles at different time points (433), again with a limited time resolution.

The assessment of circadian alignment can be obtained from the simultaneous examination of the 24-hour profiles of hormones originating from different organs. An example is the alignment of the melatonin rhythm with the leptin rhythm, reflecting the alignment of the central pacemaker with that of the adipocyte clock. A more global approach consists of examining the number of cycling genes in a given tissue. For example, in mice, sleep restriction, by reducing the amplifying and stabilizing effect of sleep on the central circadian signal, leads to an approximately 80% reduction in the number of transcripts that display circadian oscillations in the brain and in a profound disruption of the liver transcriptome. Similarly, in humans, abnormal timing of sleep causes 97% of rhythmic genes to become arrhythmic (434). This approach, although labor-intensive and costly, could be used to examine circadian alignment in conditions where the 24-hour rhythm of cortisol is disturbed or absent, as well as the efficacy of chronopharmacological replacement.

## VIII. Conclusions

Among all circulating signals used by the endocrine system, GCs are remarkable for the robustness and complexity of their secretory dynamics. Contrasting with other end-products of endocrine glands controlled by the hypothalamo-pituitary axis such as T_4_ or IGF-I, blood concentration of GCs does not remain at stable levels but is constantly oscillating with both a 24-hour periodicity and an ultradian pattern of episodic release that together provide optimal anticipation of bodily needs and rapid responsiveness to stressful stimuli. Optimal effects of GCs, at the molecular as well as at the behavioral level, occur when their rhythmic release, both circadian and ultradian, is aligned with the dynamics of local circuits expressing GR and MR. In addition to the direct effects of the temporal dynamics of GC presentation to central and peripheral tissues, the 24-hour rhythm of circulating GC levels serves as an internal synchronizer of central and peripheral circadian pacemakers, stabilizing the entire circadian system of the organism. As reviewed above, the 24-hour profile of cortisol release is disrupted in a wide variety of human conditions including misalignment with the behavioral rest-activity cycle (shift work, jet lag), dampening of the amplitude due to a failure to suppress secretion at the habitual nocturnal nadir levels (aging, sleep disorders, depression, and others), arrhythmic pattern due to autonomous hypersecretion (pituitary adenoma, adrenal adenoma, ectopic ACTH secretion, adrenal hyperplasia), and suppressed endogenous levels and rhythmicity resulting from chronic treatment with synthetic GCs or from adrenal insufficiency, either primary or secondary. The new understanding of the functional significance of the cortisol rhythm for the circadian system strongly suggests that chronopharmacological strategies that attempt to normalize the rhythm of circulating GCs need to be explored for a wide variety of physical and mental conditions and may promote disease resistance and minimize complications and adverse side effects of treatment. In conditions requiring exogenous GC administration, the timing of drug treatment should attempt to minimize the disruption of the control of circulating cortisol levels by the central circadian pacemaker. Further understanding of the mechanisms involved in the synchronization of peripheral clocks by the cortisol rhythm may lead to novel therapeutic approaches.
